# Scoping review on population pharmacokinetics of vancomycin in non-critically ill

**DOI:** 10.12688/f1000research.128260.1

**Published:** 2022-12-13

**Authors:** Diego Nivia, Juan-David Vivas, Wilson Briceño, Daniel Parra, Diego Jaimes, Rosa Helena Bustos

**Affiliations:** 1 Department of Pharmacology, Evidence-based Therapeutic Group, Faculty of Medicine, Universidad de La Sabana, Clinica Universidad de La Sabana, Chía, Cundinamarca, 140013, Colombia

**Keywords:** Population pharmacokinetic, vancomycin; non-critically patients.

## Abstract

**Background**: Vancomycin is an effective first-line therapy in MRSA infection, however, achieving an appropriate serum concentration is challenging. Population pharmacokinetics can assist the clinician in the selection of better regimen dosing and improve effectiveness and safety outcomes. **Methods:** This scoping review aims to outline the evidence in population pharmacokinetic models in non-critical adults hospitalized from 1980 to 2021 and describe the principal software and covariables used in this. A total of 209 papers were fully screened. Finally, we included 17 articles conducted in different locations around the world. **Results:** This review identified 13 retrospective articles and 4 prospective, 5 describing the use in a general population with gram-positive bacterial infection, 11 evaluated special populations (older, obese, and cancer patients), and 1 mixed population. The main parameters in the models were renal clearance and volume of distribution. The principal covariables that affected the models were creatinine clearance and weight. All studies use internal validation methods, and three of them used an external validation group. This scoping review highlights the principal information of different population pharmacokinetic models and the heterogeneity in the parameters and methods of evaluation. **Conclusions:** These methods can be used to guide the dosing regimen in different subpopulations. However, it is imperative to define the best fit in every population and conduct an experiment due to the high variability in the present studies.

## Introduction

Vancomycin is a tricycle glycopeptide antibiotic derived from *Streptomyces Orientalis*, first used in 1958. This antibiotic has become the first line in the treatment and prophylaxis of resistant gram-positive bacterial infections, especially methicillin-resistant *Staphylococcus aureus* (MRSA) and other infections such as *Clostridium difficile* infections ^1^^,^^2^ *.* Despite being highly effective, having good extraction, purification and manufacturing processes, and safe administration protocols, Vancomycin still has an important rate of reported adverse events, particularly nephrotoxicity. The rate of these adverse events is reported in different studies between 5% and 43% and is related to high doses or exposure levels, mostly in special populations like older and critical patients ^3^^,^^4^; thus, Vancomycin is considered a narrow therapeutic (NT) window drug mostly in special populations. One approach to reduce these adverse events is therapeutic drug monitoring (TDM). ^5^ Currently, an area under the curve over minimum inhibitory concentration ratio (AUC/MIC) greater than 400 mg∙h/L ^6^is considered an appropriate pharmacokinetic-pharmacodynamic (PK/PD) target. To minimize the risk of nephrotoxicity and maximize the bactericidal effect in MRSA infections, AUC/MIC should be kept between 400 and 600. ^7^

Within the different existing methods, population pharmacokinetics (PopPK) is a methodology for the characterization of pharmacokinetic parameters in target populations, furthermore PopPK has been used successfully in sparse sample data sets with 1-2 samples per patient. PopPK combines PK parameters and statistical models in specialized software that can be used to guide drug development, investigators, and clinicians on therapeutic individualization. ^7^^–^^9^ Sex had no influence in the variability of PK parameters

Regardless of being described more than 30 years ago, PopPK is not used widely because of the complexity and variety of the information. This review aims to summarize the main PK model and describe the principal software, parameters, and covariables in non-critical patients to describe the existing evidence related to PopPK models of vancomycin in hospitalized adults’ patients.

## Methods

We developed and performed a scoping review of existing literature about PopPK models of vancomycin in some populations. The research protocol was reviewed and approved by the research subcommittee of School of Medicine of Universidad de La Sabana. The review follows the manual methodology published by the Johanna Briggs Institute and the Preferred Reporting Items for Systematic Reviews and Meta-Analyses (PRISMA-ScR) ^10^^,^^11^ (see PRISMA-ScR checklist ^12^). Using the PICO framework, the following research question was formulated: “What is the existing evidence related to PopPK models of vancomycin in non-critical hospitalized adult patients?" Search criteria were established, to include studies with: (1) original models of PopPK, (2) adult patients, and (3) non-critical patients, the articles were excluded if they: (1) contained non-human studies, (2) contained incomplete studies, or (3) used other antibiotics. A search was conducted on November 23, 2021, in PubMed, LILACS, OVID Medline, Scopus, Web of Science, SAGE Journals, and Google Scholar, including original articles, reviews, systematic reviews and meta-analyses published between January 1980 and November 2021. Search terms submitted into each database are presented in Table 1. Only articles published in English, Spanish, or Portuguese were included in the search.

**Table 1.** Search constructs.

Database	Search terms
PubMed	“Population Pharmacokinetic*” [TiAb] AND (“Vancomycin“[Mesh]) AND “Adult“[Mesh] NOT “critical” NOT “children” NOT “intensive care”
OVID Medline	((population pharmacokinetic* and vancomycin and adult*) not critically).m_titl.
LILACS	((population pharmacokinetic) AND (vancomycin) AND (adult)) AND NOT (children) AND NOT (neonate) AND NOT (pediatric) AND NOT (critically) AND NOT (intensive care)
Web of Science	(((TI= (population pharmacokinetic*)) AND TI=(vancomycin)) AND TI=(adult)) NOT TI=(critically)
SAGE Journals	[Title population pharmacokinetic*] AND [Title vancomycin] AND [adults] AND NOT [Title critical]
Scopus	TITLE (population AND pharmacokinetics) AND TITLE (vancomycin) AND (adult) AND NOT critical AND NOT pediatric
Google Scholar	allintitle: population pharmacokinetic vancomycin adult -critically

Search results were uploaded into Rayyan software, ^13^ and the duplicates were eliminated. Titles and abstracts were organized and screened by two authors (J.-D.V. and D.N.) using Rayyan software. Articles that did not meet inclusion/exclusion criteria were removed. The selected articles were reviewed by two authors (J.-D.V. and D.N.) and compared to inclusion and exclusion criteria. There were no disagreements between the two authors. The results were categorized and organized into subgroups in Table 2 and Table 3.

**Table 2.** Summary of Demographics and PopPK modeling methods for all the studies.

	Article	Year	Country	Study design	Population	Sample size, (male/female)	Age (years), mean (SD)	Weight (kg), mean (SD)	BMI	N.° compartments	Software	Validation
**GENERAL**	Deng C *et al.* ^14^	2013	China	Retrospective	Adult patients	72 (19/53)	54.07 (18.36)	61.12 (10.70)	NR	One compartment	NONMEM® version 7.2	Internal: Bootstrap (n=2000), VPC
	Ji XW *et al.* ^15^	2018	China	Retrospective	Patients who received continuous infusion of vancomycin and were not on renal replacement therapy	160 (106/54)	78 (42-95) range	65 (38-90) range	22.31 (12.85–36.89) range	One compartment	NONMEM® version 7.3	Internal: Bootstrap (n=1000) and NPDE; External validation (n= 58)
	Medellín-Garibay SE *et al.* ^16^	2015	Spain	Retrospective	Adult patients from the Traumatology Service with proven or suspected infection	118 (53/65)	74.3 (14)	72.0 (15)	27.5 (5)	Two compartments	NONMEM® version 7.2	Internal: Bootstrap (n=200); External validation: ( n=40)
	Yamamoto M *et al.* ^17^	2009	Japan	Retrospective	Adult patients with a suspected or documented infection caused by gram-positive bacteria.	106 [(100 patients (64/36), 6 healthy subjects) (6/0)]	Healthy subjects: 21.7 (20-25) range Patients: 65.4 (25.8-99.7) range	Healthy subjects: 60.3 kg 55.2-64.2) range Patients: 52.6 Kg (28.7-97)	NR	Two compartments	NONMEM® version 5.1	Internal: Bootstrap
	Yasuhara *et al.* ^18^	1998	Japan	NR	Hospitalized patients infected with MRSA.	190 (131/59)	64.3 (13.8)	52.3 (9.6)	NR	Two compartments	NONMEM® version 7	goodness-of-fit plots model.
	Tanaka *et al.* ^19^	2010	Japan	Prospective	Patients with MRSA infections and who were receiving Vancomycin treatment	164 (104/60)	74 (17-95) range	53 (10)	NR	One compartment	NONMEM® version 5	MAE
	Alqahtani *et al.* ^20^	2020	Arabia Saudi	Retrospective	Adult patients older than 18 years old with cancer and non-cancer.	74 (44/30)	55.1 (15.9)	75.5 (19.7)	27.1 (5.8)	One compartment	Monolix® version 4.4	Internal: (pcVPC)
**RENAL**	Kim DJ *et al.* ^21^	2019	South Korea	Retrospective	Patients with vancomycin treatment for various infections, and at least two serum concentration measurements	99 (59/40)	64.8 (12.6)	59.7 (10.98)	22.30 (3.93)	Two compartments	NONMEM® version 7.4	Internal: Bootstrap (n=1000)
	Ma Kui-fen *et al.* ^22^	2020	China	Retrospective	Patients who received vancomycin as prophylactic medication following kidney transplant operation	56 (35/21)	43.72 (9.92)	58.27 (8.47)	NR	One compartment	NONMEM® version 7.4	goodness-of-fit plots model.
	Pai M P. *et al.* ^23^	2020	USA	Retrospective	Patients with stable and unstable kidney disease	2640 (1689/950)	59 (16)	93.9 (28.1)	31.7 (9.0)	One compartment	Monolix® 2019R2	Internal: Bootstrap (n=1000)/(NPDE)
	Schaedeli *et al.* ^24^	1998	Switzerland	Retrospective	Patients undergoing long term hemodialysis who received vancomycin for infection therapy or prophylaxis	26 (16/10)	62 (15.2)	Dry weight: 64.7 (13.6)	NR	Two compartments	NONMEM®	Internal: prediction models?
OBESE	Adane *et al.* ^25^	2015	USA	Prospective	Extremely obese adult patients (BMI ≥ 40 kg/m2) with suspected or confirmed Staphylococcus aureus infection	31 (19/12)	43 (38.5-53) range	147.6 (142.8-178.3) range	49.5 (44.3–54.8) range	Two compartments	NONMEM® 7.3	Internal
**GERIATRICS**	Sanchez *et al.* ^26^	2010	USA	Retrospective	Adult and geriatric patients	141 (NS)	55 (14.58)	73.2 (17.48)	NR	Two compartments	NONMEM® version VI	Internal: Bootstrap (n=200)
	Zhou *et al.* ^27^	2019	China	Retrospective	Elderly patients (age ≥65 years) with HAP or CAP	70 (49/21)	78.3 (6.96)	60.7 (10.2)	NR	One compartment	NONMEM® version 7.3.0	Internal: Bootstrap (n=1000) and NPDE
	Zhang et al ^28^	2020	China	Prospective	Elderly patients (age ≥65 years) infected	150 (104/46)	73.6 (6.83)	61.7 (1 1.1)	NR	One compartment	NONMEM® version 7.4	Internal Bootstrap (n=2000) and NPDE
**CANCER**	Alqahtani *et al.* ^20^	2020	Arabia Saudi	Retrospective	Adult patients older than 18 years old with cancer and non-cancer.	73 (58/42)	53.8 (15.7)	72.7 (16.2)		One compartment	Monolix® version 4.4	Internal: (pcVPC)
	Santos-Buelga *et al.* ^29^	2005	Spain	Retrospective	Adult (15-year-old) in patients with an underlying hematological malignancy admitted for suspected or documented infection caused by gram-positive bacteria	215 (119/96)	51.5 (15.9)	64.7 (11.3)	NR	One compartment	NONMEM® version 5.1.1	Internal
	Okada *et al.* ^30^	2018	Japan	Retrospective	Patients undergoing allo-HSCT who received preventive treatment with vancomycin	75 (49/26)	49 (17-69) range	59.4 (39.4-104.5) range	NR	Two compartments	Phoenix NLME® 7.0	Internal: Bootstrap (n=1000); external validation (20 patients)

NR= No reported NS= No specified MRSA= Methicillin resistant *Staphylococcus aureus* USA=United states

**Table 3.** Characteristics PK models.

	Author	Clearance related parameters: CL (L/h), Q (L/h), k (h ^-1^)			Volume related parameters V (L), V2 (L)			BSV		RV	
		Formula	Parameter	Value	Formula	Parameter	Value	CL	V	Proportional	Additive (mg/L)
**GENERAL**	Deng C *et al.* ^14^	CLCR<80 mL/min: CL=θ _1_×CLCR CLCR≥80 mL/min: CL=θ _2_	θ _1_ θ _2_	0.0654 4.9	V = θ _3_	θ _3_	47.76	45.35 %	39.25 %	30.71%	1.21
	Ji XW *et al.* ^15^	CL = θ _1_ × (1+θ _2_ × [CLCR- 80]) ×(75/AGE) ^ θ _3_	θ _1_ θ _2_ θ _3_	2.829 0.00842 0.8143	Vd= θ _4_	θ _4_	52.14	32.42 %	28.87 %	26.79%	2.64
	Medellín-Garibay SE *et al.* ^16^	Furosemide=0: CL = θ _1_ × CLCR Furosemide=1: CL = θ _5_×CLCR Q = θ _3_	θ _1_ θ _5_ θ _3_	0.49 0.34 0.81	V1 (L/kg) =θ _6_×TBW (if age ≤65 years) V1 (L/kg) =θ _2_×TBW (if age > 65 years) V2 (L/kg)= θ _4_×TBW	θ _6_ θ _2_ θ _4_	0.74 1.07 5.99	CL=36.2%	V1= 37.1%	19.3%	
	Yamamoto M et al ^17^	CLCR>85 mL/min: CL = θ _1_ CLCR<85 mL/min: CL = θ _2_ x CLCR + θ _3_ Q=θ _8_	θ _1_ θ _2_ θ _3_ θ _8_	3.83 0.0322 0.32 8.81	V1 = θ _4_ x (1+(θ _5_ x STATUS ^a^)) x WT V2 = θ _6_ + (STATUS ^a^ x θ _7_)	θ _4_ θ _5_ θ _6_ θ _7_	0.206 0.272 39.4 21.2	CL=37.5% Q=19.2%	V1=18.2% V2= 72.8%	14.3%	
	Yasuhara *et al.* ^18^	CLCR ≤ 85 mL/min: CL=θ _1_ x CLCR CLCR >85 mL/min: CL=θ _2_ k12= θ _3_ k21= θ _4_	θ _1_ θ _2_ θ _3_ θ _4_	0.0478 3.51 0.525 0.213	Vss= θ _5_	θ _5_	60,7	CL=38.5% k21=25.4%	Vss= 25.4%	23.7%	
	Tanaka *et al.* ^19^	CL (ml/min) = θ _1_ x GFR	θ _1_	0.875	V (L/kg)= θ _2_	θ _2_	0.864	19.8%	30.7%	12.7%	
	Alqahtani *et al.* ^20^	CL= θ _1_ x (CLCR/96.3)^θ _2_	θ _1_ θ _2_	5.6 0.18	V= θ _3_	θ _3_	42	20.3%	18.2%	23%	
**RENAL**	Kim DJ *et al.* ^21^	CL= θ _1_ × [(θ _2_/baseline of GFRindividual) + (GFR at time/GFRmedian)] Q = θ _5_	θ _1_ θ _2_ θ _5_	2.21 0.921 3.06	V1 = θ _3_ V2 = θ _4_	θ _3_ θ _4_	32.6 45.8	CL=5.3% Q=70.9%	V2 = 32%	14.3%	1.95
	Ma Kui-fen *et al.* ^22^	CL= θ _1_ x [(WT/59.95)^θ _2_]x[(GFR/36.67) ^θ _3_]	θ _1_ θ _2_ θ _3_	2.08 0.698 1.07	V= θ _4_ x [(WT/59.95)^θ _5_]	θ _4_ θ _5_	63.2 0.934	21.5 %		24.2%	
	Pai M P. *et al.* ^23^	CL = exp(θ _1_ + θ _2_ x (eGFR/100)) - θ _3_	θ _1_ θ _2_ θ _3_	1.03 0.737 -1.63	V1= θ _4_	θ _4_	66.4	θ _1_= 1.82 θ _2_= 1.24 θ _3_= 1.32			0.76
	Schaedeli *et al.* ^24^	CLCR ≥2 mL/min: CL= θ _1_+ θ _2_ x CLCR CLCR< 2 mL/min: CL = θ _1_ CLDv= θ _3_ x CLD _BUN_ K12 = θ _5_ K21 = θ _6_	θ _1_ θ _2_ θ _3_ θ _5_ θ _6_	2.25 0.585 0.336 0.872 0.162	V(L) = θ _4_ x WT	θ _4_	0.164	CLCR <2 mL/min: Cl= 90% CLCR ≥2 mL/min: Cl= 32% CLDv = 13%	V= 22%	13%	
**OBESE**	Adane *et al.* ^25^	Cl = θ _2_ x (ClCR/125)	θ _2_	6.54	V = θ _1_ x TBW	θ _1_	0.51	26.70 %	23.90 %	18.9 %	
**GERIATRICS**	Sanchez *et al.* ^26^	CL= θ _1_+θ _5_ × CLcr Q = θ _4_ x TBW	θ _1_ θ _5_ θ _4_	0.157 0.563 0.111	V1 = θ _2_ × TBW V2 = θ _3_ × AGE/53.5	θ _2_ θ _3_	0.283 32.2	CL = 24.49 %	V2= 6.8 %	24.9 %	
	Zhou *et al.* ^27^	θ _1_×(CLCR/56.28) ^ θ _2_	θ _1_ θ _2_	2.45 0.542	V1 = θ _3_	θ _3_	154	CL=17.53%	V=34.90%	6.57 %	
	Zhang *et al.* ^28^	CL= θ _1_ x (GFR/80)^ θ _2_ x (1 + θ _3_ x PCM)	θ _1_ (L/h) θ _2_ θ _3_	3.74 1.03 0.41	V1= θ _4_	θ _4_ (L)	118	CL= 44.26%	V= 54.99%		0.184 (log scale)
**CANCER**	Alqahtani *et al.* ^20^	CL= θ _1_ x (CLcr/99.9) ^ θ _2_	θ _1_ θ _2_	7.4 0.21	V = θ _3_	θ _3_ (L)	45	15.9%	13.8 %	12.5%	
	Santos-Buelga *et al.* ^29^	CL = θ _1_ x CLCR	θ _1_	1.08	V=θ _2_ x TBW	θ _2_	0.98	28.16 %	37.15 %		3.52
	Okada *et al.* ^30^	CL = θ _2_ × (CLCr/113) ^ θ _6_ Q = θ _4_	θ _2_ (L) θ _6_ θ _4_	4.25 0.70 1.95	V1 = θ _1_ × (BW/59.4) ^ θ _5_ V2 = θ _3_	θ _1_ (L/h) θ _5_ θ _3_ (L)	39.2 0.78 56.1	25.2 %	V1= 14.2 % V2=66.9 %	17.2 %	

BSV= between-subject variability, BUN = blood urea nitrogen, CL= Clearance, CLcre= creatinine clearance, CLD _BUN_= in vivo urea dyalisis clearance, GFR= glomerular filtration rate, NS= No specified, PCM= post-craniotomy meningitis, RV= residual variability, TBW= total body weight, Vc=central volume, Vp= plasma volume, Vd= volume of distribution, V (ss)= steady-state volume of distribution, WT= weight

^a^ STATUS: Value 1 for patients with gram-positive infections.

## Results

**Figure 1.**
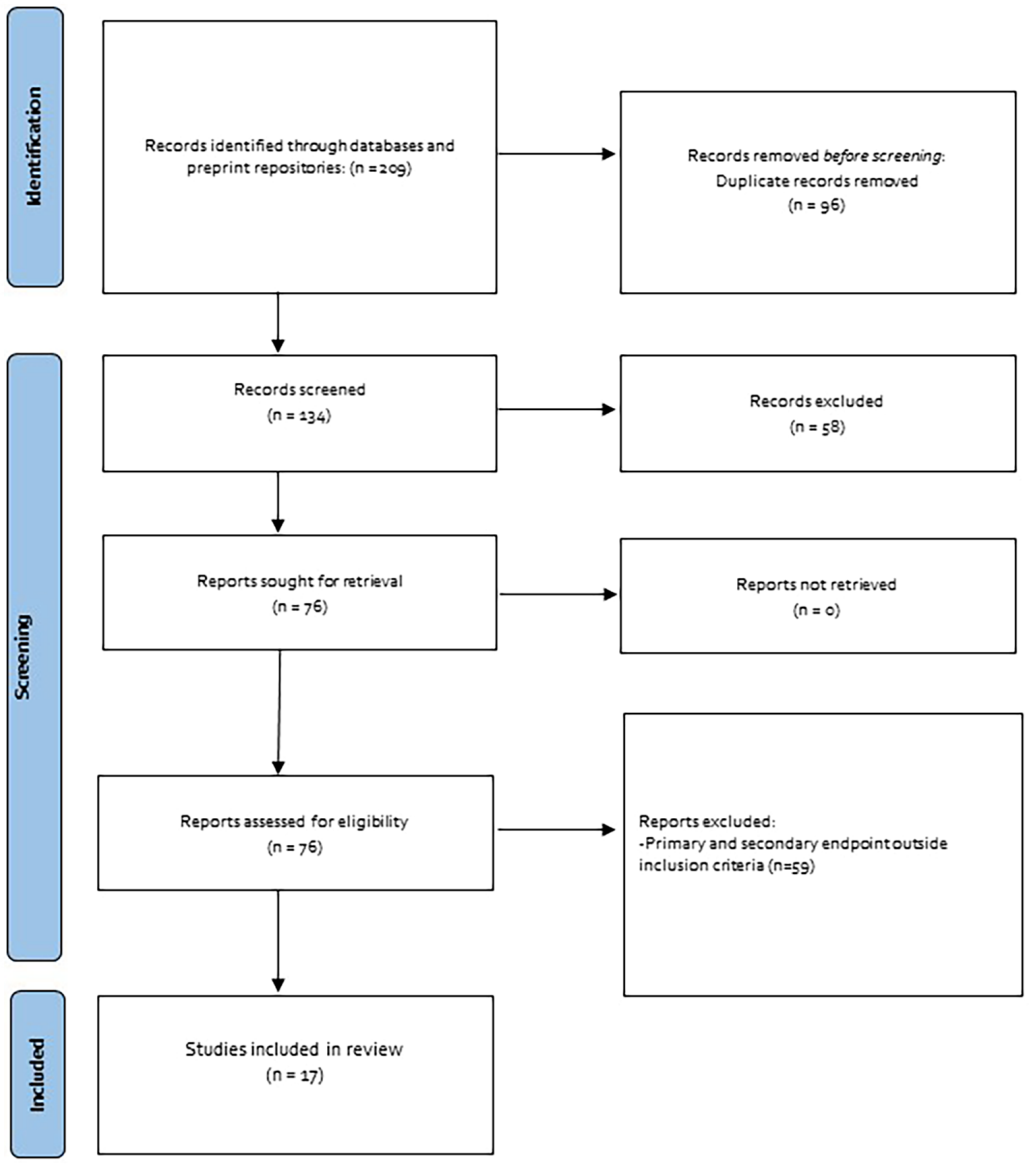
Flowchart of studies selected.

Searches were conducted in November 2021 and 209 articles were identified in the selected databases. After the exclusion of duplicates, 134 articles remained for screening and 73 of these articles were selected for review. Finally, we considered 17 articles that met the inclusion/exclusion criteria. 11 articles were conducted in Asia, 3 in Europe, and 3 in the USA. 4 studies did not specify the clinical characteristics of the subjects and 1 has a mixed population of cancer and non-cancer patients. The study populations were categorized into four groups: (a) renal, (b) obese, (c) older, and (d) cancer patients ( Figure 1).

13 of the 17 articles were retrospective, 3 were prospective, and 1 had an unreported temporality. The number of patients varied widely among the studies, from 26 in the study of Schaedeli *et al.* ^24^ to 2640 patients in the study of Pai *et al.*. ^23^ The mean age and weight were 60 years (SD 12.64 years) and 59 kg (SD 6 kg), respectively; these results are shown in Table 2.

Most of the studies reported a one-compartment model (N = 10) as the final model, while the rest of the studies reported a two-compartment model (N = 7). Only the study by Schaedeli *et al.* ^24^ reported an additional route for drug elimination consisting of dialysis removal. Regarding modeling software, 14 articles (82.3%) used NONMEM (Nonlinear Mixed Effects Modelling) software, 2 articles used Monolix, and 1 article used Phoenix.

All the studies had internal validation, the most common method was bootstrapping but some studies showed visual predictive checks, residuals and goodness-of-fits plots, as well as other measures to assess predictive performance. Only 3 studies (17.3%) had external validation ( Table 3).

Table 3 summarizes the results of the 17 studies. The two PK parameters present in all models are clearance (CL) and volume of distribution (V). Most of the studies did not show the typical value and presented the results in varying measurement units. When we calculated the typical value of all included studies, the results were highly variable. The mean value of the estimated CL in the different studies was 3.76 ± 1.63 L/h.

We calculate the typical values of the distribution of pharmacokinetic parameters (TV) with the aim of improving comparison among studies. This calculation was done with measures of central tendency for the reported covariates and substituting them in the covariate equations in the final model. The median of typical values for vancomycin clearance (TVCL) among studies was 3.80 (interquartile range 2.84 to 5.42) L/h, and the range for CL was 2.08 to 7.48 L/h (see additional results tables ^12^).

The study with the highest value of the vancomycin CL was the one performed by Pat MD *et al.*, ^23^ in this study the TVCL was 7.48 L/h. The population explored in this study were patients with non-stable kidney disease, in these patients a mean CLCR value of 100mL/min was determined. The lowest vancomycin CL value was reported in the study by Ma KF *et al.*, ^22^ where a typical value of 2.08 L/h was reported. For the patients in this study, a mean value of 2.08mL/min was reported for CLCR.

Some important differences were observed for TVCL when considering subpopulations. For cancer patients there were higher CL values with a median of 5.79 (interquartile range 5.02 to 6.60) L/h compared to general patients with a median TVCL of 3.69 (interquartile range 2.85 to 4.38) L/h. Although there was only one study in obese patients, this had a high TVCL compared to the general patient subpopulation with TVCL equal to 6.54 L/h.

In the studies performed on populations with kidney disease, there was a wide variability in the value of TVCL with a median of 3.54 (range 2.08 to 7.48) L/h, which could be related to the different states of glomerular filtration in the studies. Finally, geriatric patients presented low TVCL values, although they were not very different from those presented in general population.

This clearance is estimated using creatinine, except in one study in the Japanese population. In this article, they used cystatin C as a renal marker in a one-compartment model. ^19^ High estimates were observed in extremely obese and cancer patients ^20^^,^^25^ and lower values in geriatric and renal patients. ^27^^,^^31^

The estimated typical values of volume of distribution (TVV) were highly variable, with median of 57.7 (interquartile range 46.9 to 65.7) L and range from 42 to 154 L for one-compartment models. The lowest TVV was 42 L in the subgroup of non-cancer patients in the Alqhatani study ^20^ and the highest was 66.4 L in the patients with unstable and stable kidney function. ^7^ In elderly patients, the estimated values of TVV with median of 136 (interquartile range 127 to 145) L were higher compared to general patients’ median of 47.2 (interquartile range 45.5 to 48.9) L.

The median of typical values for total volume (TVV _TOTAL_), defined as the sum of volumes in the reported compartments, among studies was 63.5 (interquartile range 52.3 to 91.1) L, and the range for V _TOTAL_ was 10.6 to 508 L. In the case of two compartment models, the highest estimated TVV _TOTAL_ was 508.32 L in the Spanish study of trauma patients ^16^ and the lowest was 60.7 L in the Japanese population. ^17^ For TVV _TOTAL_, there were also high values in the geriatric population compared to the general population.

Although most studies with two-compartment models reported parameters in the form of flow rates (CL and Q), two studies reported model parameters in the form of elimination, transfer rate constants (k _10_, k _12_, k _21_) were presented. In order to make comparisons among studies, the conversion of parameters in the form of flow rates was implemented with the following relationships: CL=k _10_*V _1_, Q=k _12_*V _1_, V _2_=Q/k _21_.

The typical values of intercompartmental clearance (TVQ) were variable among two-compartment models, with median of 8.13 (interquartile range 2.50 to 9.03) L/h. There were no apparent differences among the studied population subgroups.

The between-subjects variability was reported in most articles as a percentage. The highest between-subject variability (BSV) values were observed in a BSV estimation on V2 with a coefficient of variation (CV) of 72.8%. ^17^ The residual variability was stated as combined in additive proportional in 3 articles and is present in additive terms in five articles; the type of error was not found in one article.

The main covariables used in the different models were estimated glomerular filtration rate, weight, and, in some cases, age. One article used furosemide and other cystatin C for glomerular filtration rate (GFR) estimation. GFR was the main covariable that affected the models, and this covariate was used to explain between-individual variability in drug clearance.

## Discussion

The development of population pharmacokinetics within the precision medicine measures is relevant to ensuring adequate therapy in special populations and monitoring drug therapy with a narrow therapeutic index. Biosimulation helps to improve efficacy and decrease toxicity based on the covariables that impact the drug's pharmacokinetics. In the case of vancomycin, software such as NONMEM could be used to calculate AUC and monitor therapy according to the most recent ASHP vancomycin monitoring guidelines. However, this technology requires clinical pharmacology experts and is limited to university centers and research sites in most cases. ^7^^,^^32^

In this review, the NONMEM software analysis (originally developed at the University of San Francisco in 1978) was the most frequently used in the articles. This program is the oldest and is considered the standard, while Monolix (Lixoft, Paris, France) and Phoenix NLME (Certara, Princeton, NJ) were released more recently and were less frequently used. ^33^ All three are commercial offerings with substantial license fees, and although all have programs intended to reduce or eliminate licensing costs in educational institutions, low-income countries, or both, the administrative hurdles and associated delays in availability can be cumbersome when running analyses and training students and researchers to use these tools in resource-limited settings. ^33^ Some other factors may influence the selection of software, such as the differences between NONMEM and Monolix. NONMEM has implemented new algorithms in his software, but the run time seems to be very long compared to Monolix. Phoenix, for its part, is the cheapest of the three.

Vancomycin is excreted 80-90 % as an unchanged drug in urine, ^34^ for that reason, it is expected that creatinine clearance would be the most important covariable in most models and could affect the prediction of serum vancomycin concentration. Despite the multiple limitations of the Cockcroft Gault equation, most of the articles used it; however, some models show that the CKD-EPI (Chronic Kidney Disease Epidemiology Collaboration) equation is more accurate, especially in older populations and one of the papers uses cystatin C, a renal marker that is considered more accurate and sensible than creatinine, which suggests that it could be a good predictive marker of serum vancomycin levels. ^15^^,^^19^

An important observation in the parameters of the structural model (volume and clearance) was that some populations were above the mean value of the data. In the volume parameter, we can notice that the two volumes that are above the mean are those of the geriatric patients and the general group. In the first group, this finding is constant in the different pharmacokinetic studies; this may be due to changes in the peripheral circulation and increased tissue affinity for vancomycin; ^35^ in the second, it is due to the elevated V2 of the patients of the traumatology service of the Medellin Garibay article. This is a sign of overparameterization of the model in possible relation to its design due to the small number of samples of each patient. ^16^

In the clearance parameter, the populations that are above the average are the obese, this is explained by the hyperfiltration resulting from compensatory vasodilation of the afferent arteriole ^36^ and cancer patients, which is associated with hyperdynamic circulation caused by systemic inflammation and direct cytokine activation of renal cation and anion transporters. ^20^

Another significant finding was that race or origin site had no significant influence on the models in the various studies. For the obese subpopulation the elimination rate constant (k _10_=CL/V) could be affected by the patients being overweight and in the geriatric subpopulation by change in the central compartment volume. A solution to this problem would be to increase the loading dose to achieve a faster steady state and make it easier for them to achieve the target of AUC/MIC > 400 to ensure an adequate bactericidal effect.. ^17^^,^^18^^,^^25^^,^^26^ Despite this, CLCR, as in other populations, seems to be the covariate that most influences CL. ^27^^,^^28^

In patients with kidney disease, the CKD-EPI equation appears to be more accurate than MDRI and CG. ^21^^,^^23^ Kidney disease population, the one-compartment model seems to be better in patients with unstable renal function and transplant patients. In patients with unstable renal function, using a single point-in-time measure may be less reliable for dose changes, so a time-varying model varying covariate structure was superior to a time-invariant one. ^23^ The two-compartment models appear to overestimate, and the only subpopulation that appears to fit better with the two-compartment models is intermittent dialysis patients, owing to a rebound in serum vancomycin concentrations after intermittent dialysis. The heterogenicity of this population due to changes in the central compartment generated by dialysis and changes in the ultrafiltration rate of each session is very high, and CLCR is not a reliable marker of renal function. ^24^

Finally, cancer patients are another interesting group for populational pharmacokinetics. There is evidence of suboptimal antibiotic therapies about the hyperdynamic state caused by systemic inflammation ^37^ and activation of renal cationic transporters by cytokines. ^38^ This state generates an increased renal CL, causing this population to require higher maintenance doses to maintain AUC/MIC > 400, especially during periods of neutropenia. ^20^^,^^29^ For patients undergoing allogeneic transplantation, the models developed in this population indicate a high variability due to high between-subject variability and the difficulty of maintaining the therapeutic range due to the characteristics of these patients with extremely low Hematocrit levels, increased intravascular volume, and increased renal clearance. ^29^^,^^30^

This review had several limitations. Some of the papers do not specify the clinical and pathological characteristics of the study subjects. The creatinine clearance formulas are different in every article making necessary the classification of every subpopulation before applying the model, the units of measure and the population have great variability. That is the main reason why the comparisons presented are indirect and the generalization of the data that we show must be read carefully. Several studies did not report the probability of reaching the AUC/MIC target >= 400. This was because the value of AUC/MIC >= 400 was defined as the optimal PK/PD “efficacy” target value for the bactericidal effect of vancomycin, years after these studies.

## Conclusions and recommendations for future research

This scoping review highlights the principal information of different population pharmacokinetic models and the heterogeneity in the parameters and methods of evaluation. The principal modeling software reported in the articles is NONMEM; however, it wasn’t possible to evaluate the external validation in the PK model reported in this article, and important differences were also found in the set of PK parameters reported within the subpopulations found. Even if these methods can be used to guide the dosing regimen in different subpopulations, it is imperative to conduct experiments with local samples and patients to define the best fit in the different subpopulation.

## Data Availability

All data underlying the results are available as part of the article and no additional source data are required. Zenodo: Scoping review on Population pharmacokinetics of vancomycin in non-critically ill. https://doi.org/10.5281/zenodo.7296549 ^12^ This project contains the following underlying data:
‐ PkPop Vanco_non critical patients - Extended data E1 PRISMA-ScR checklist.docx ‐ PkPop Vanco - Extended data E2 Additional results tables.pdf Data are available under the terms of the Creative Commons Attribution 4.0 International license (CC-BY 4.0).
