# Vancomycin Population Pharmacokinetic Models in Non- Critically Ill Adults Patients: a scoping review

**DOI:** 10.12688/f1000research.128260.2

**Published:** 2025-03-06

**Authors:** Diego Nivia, Juan-David Vivas, Wilson Briceño, Daniel Parra, Manuel Mena, Diego Jaimes, Juan-Francisco Guevara, Rosa Helena Bustos

**Affiliations:** 1 Department of Pharmacology, Evidence-based Therapeutic Group, Faculty of Medicine, Universidad de La Sabana, Clinica Universidad de La Sabana, Chía, Cundinamarca, 140013, Colombia

**Keywords:** Population pharmacokinetic, vancomycin; non-critically patients.

## Abstract

**Background:**

Vancomycin is an effective first-line therapy primarily in methicillin-resistant Staphylococcus aureus (MRSA) infection and Clostridium difficile, however, it has been shown that its effectiveness and the reduction of nephrotoxicity depend on maintaining adequate therapeutic levels. Population pharmacokinetic (PopPk) models attempt to parameterize the behavior of plasma concentrations in different target populations and scenarios such as renal replacement therapy, to successful therapeutic outcome and avoid these side effects.

**Methods:**

A scoping review was conducted following the guidelines of Preferred Reporting Items for Systematic reviews and Meta-Analyses Extension for Scoping Reviews (PRISMA-ScR), through a search in PubMed, LILACS, OVID Medline, Scopus, Web of Science, SAGE Journals, Google Scholar and previous known registers of PopPk models in non-critically ill adult patients, published between 1998 and 2024.

**Results:**

A total of 190 papers were fully screened, of which were included 36 studies conducted in different populations; 12 in general population, 23 in special populations (surgical, with impaired renal function, obese, elderly, with cancer and cystic fibrosis), and 1 in mixed population (general and with cancer). The main parameters in the models were renal clearance and volume of distribution. The principal covariables that affected the models were creatinine clearance and weight. All studies used internal evaluation and 4 of them used an external group.

**Discussion:**

The technology for the development and implementation of PopPk models requires experts in clinical pharmacology and is limited to university and research centers. The software is mostly expensive and, in most cases, the pharmacokinetic models and the heterogeneity in the parameters and evaluation methods depend on which compartmental model, parameters, covariates and software have been used.

**Conclusions:**

These models require validation in the clinical context and conducting experiments to adapt them for precision dosing in different subpopulations.

## Introduction

Vancomycin is a tricycle glycopeptide antibiotic derived from *Streptomyces orientalis*, first used in 1958; by inhibiting the synthesis of the wall, it achieves a high bactericidal power against methicillin-resistant *Staphylococcus aureus* (MRSA), methicillin-susceptible *Staphylococcus aureus* (MSSA), streptococci, enterococci and *Clostridium difficile*. ^1^^,^^2^ Pharmacokinetic and pharmacodynamic studies of vancomycin suggest that prior trough monitoring is associated with increased nephrotoxicity, with rates between 5% and 43%, related to high doses or high levels of exposure, mainly in special populations such as elderly and critically ill patients. ^3^^,^^4^ Therefore, the current dosing and monitoring recommendations of the revised consensus guideline and review by ASHP/PIDS/SIDP/IDSA establish an AUC/MIC ratio of 400–600 h ^−1^ (assuming a MIC of 1 mg/L) to achieve clinical efficacy and ensure safety for patients treated for serious MRSA infections. ^5^

Although therapeutic drug monitoring (TDM) for vancomycin remains controversial, it has been shown to significantly increase the rate of clinical efficacy and decrease the rate of nephrotoxicity. ^5^ TDM of Vancomycin is essential for the development of PopPK models by the use of Bayesian software for AUC estimation and model-informed precision dosing (MIPD), which has been improved outcomes in patients with culture-proven gram-positive infections just with a single concentration monitoring. ^6^ The PopPK modeling plays a crucial role in optimizing drug dosing regimens, particularly for drugs with a narrow therapeutic index (NTI). NTI drugs, where small changes in drug concentration can result in significant therapeutic consequences, require precise dosing to maximize efficacy and minimize toxicity. ^7^ PoPK models enable the integration of various physiological, biochemical, and drug-specific factors to predict drug behavior in different patient populations. By simulating a range of dosing scenarios, these models allow for the identification of optimal dosing strategies that balance therapeutic benefit with safety, ensuring that NTI drugs achieve their intended clinical outcomes while avoiding adverse effects. This modeling approach is essential for personalizing therapy, reducing the risk of underdosing or overdosing, and improving patient outcomes. ^8^

PopPK is an emerging discipline developed from the combination of classical pharmacokinetic compartment model and statistical principles, which helps to achieve the preliminary prediction of parameters. ^9^ Despite having been described more than 30 years ago, PopPK models are not widely used due to mathematical complexity, the variety of the study population and limited access to software. ^10^ This review aims to summarize the main models, software, parameters and covariates in non-critical adult patients that can be used in future applications for MIPD.

## Methods

We developed and performed a scoping review of existing reports about PopPK models of vancomycin in adult population out of intensive care. The research protocol was reviewed and approved by the research subcommittee of School of Medicine of Universidad de La Sabana. The review follows the guidelines of Preferred Reporting Items for Systematic reviews and Meta-Analyses Extension for Scoping Reviews (PRISMA-ScR) and the Johanna Briggs Institute. ^11^^,^^12^ Reporting checklist extended data E1.

The primary review question was formulated using the population, concept, context framework, as “What are the published vancomycin population pharmacokinetic models with non-critically ill hospitalized adult patients?”. Search criteria were established to include studies with: (1) original vancomycin PopPK models, (2) adult patients, and (3) non-critical ill patients; articles were excluded if they: (1) are the wrong publication type, (2) patients are hospitalized in the intensive care or burn unit, (3) do not define equations or parameters, and (4) have a broad range of patients, including critically ill patients. The search was conducted on November 2, 2024, in PubMed, LILACS, OVID Medline, Scopus, Web of Science, SAGE Journals, and Google Scholar, including reports published after January 1998 and some previously known reports in other similar publications. Search terms submitted to each database are presented in Table 1. Only articles published in English, Spanish, or Portuguese were included in the search. The founded references were uploaded into Rayyan ( http://rayyan.qcri.org; Headquarter: Cambridge, Massachusetts, U.S.A.), which is a free web and mobile app, that helps expedite the initial screening of abstracts and titles using a process of semi-automation while incorporating a high level of usability. ^13^ First the detected duplicates in data summary was eliminated by preliminary revision, then the reports were screened and selected for the full text screening to check over the inclusion and exclusion criteria.

**Table 1.** Search constructs.

Database	Search terms
PubMed	(("vancomycin"[All Fields]) AND ("population pharmacokinetic"[All Fields])) NOT ("critically ill"[All Fields])
OVID Medline	((population pharmacokinetic* and vancomycin and adult*) not critically).m_titl.
LILACS	((population pharmacokinetic) AND (vancomycin) AND (adult)) AND NOT (children) AND NOT (neonate) AND NOT (pediatric) AND NOT (critically) AND NOT (intensive care) AND ( mj:("Vancomycin"))
Web of Science	(((((TI=(vancomycin)) AND TI=(population pharmacokinetic)) NOT TI=(critically ill)) NOT TI=(neonates)) NOT TI=(pediatric)) NOT TI=(infants)
SAGE Journals	[Title population pharmacokinetic] AND [Title vancomycin]
Scopus	TITLE (population AND Pharmacokinetic) AND TITLE (vancomycin) AND ALL (adult) AND NOT ALL (critically AND ill) AND NOT ALL (pediatric) AND NOT ALL (children) AND NOT ALL (neonates) AND NOT ALL (infants)
Google Scholar	allintitle: population pharmacokinetic vancomycin adult-critically

## Results

**Figure 1.**
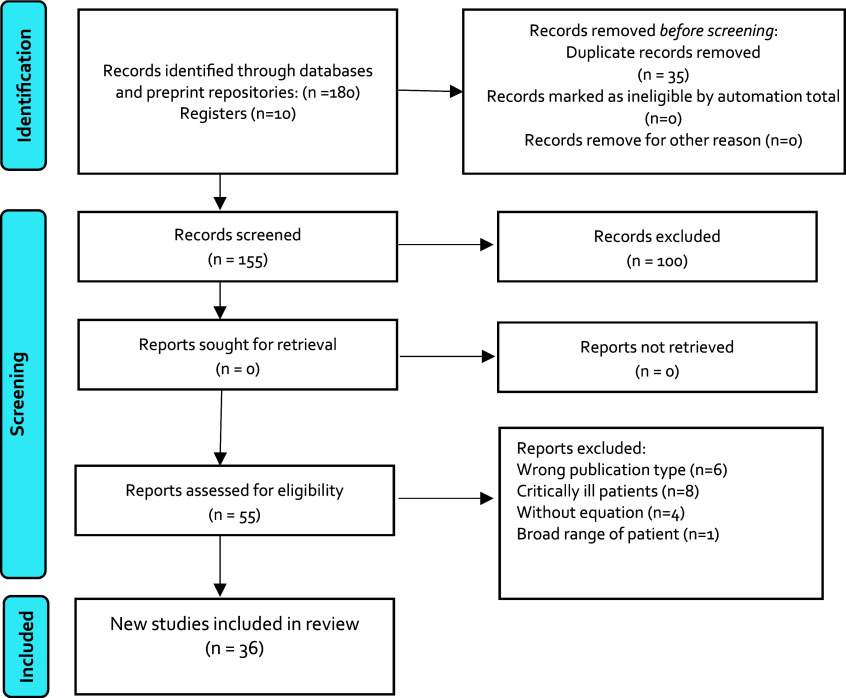
Flowchart of studies selected.

We identified 180 records in databases and 10 registers previous included in others publications. ^14^ After removing duplicates, 155 records remained for screening; 100 were eliminated due to exclusion criteria and 55 reports were assessed for eligibility for full text review; finally, 36 studies were included for this review ( Figure 1).

In order to organize the information, they were divided into 8 groups of patients, general (12), ^15^^–^^26^ surgical (5), ^27^^–^^31^ with impairment kidney function (7), ^32^^–^^38^ obese (3), ^39^^–^^41^ geriatrics (4), ^42^^–^^45^ with cancer (4), ^15^^,^^46^^–^^48^ patients with cystic fibrosis (1) ^49^ and trauma patients (1). ^50^ Studies published from 1998 to 2024 were found, the mean study by year was 2.3 with an standard deviation (SD) of 1.6 and the years with the highest number of publications were, 2018 (6), 2020 (5), 2019 (4) and 2024 (4). Predominantly, publications of Asian, Middle Eastern, North American and European origin were found; the countries with the highest number of publications were China (9), Japan (6), South Korea (5) and the United States of America (USA) (5). Regarding the design of the study, 27 (75%) were retrospective and 9 (25%) were prospective; the mean of sample size was 217 and SD of sample size was 434,4 (biased by the study of Pai and DeBacker ^36^ with 2640 patients). 22 (61.1%) of the models were one-compartment and 14 (38.9%) were two-compartment.

The most commonly used software was NONMEM in 28 studies (77.8%), followed by Monolix 5 (13.9%), Phoenix 2 (5.6%) and R environment with Pmetrics package 1 (2.8%), therefore, 32 (88.9%) of the studies performed a primary analysis and development of the model with Nonlinear mixed-effects modeling (NLME), followed by 3 (8.3%) that used stochastic approximation expectation maximization (SAEM) and 2 (2.8%) performed with nonparametric adaptive grid (NPAG); the most used secondary analyses were first-order conditional estimation (FOCE), first-order conditional estimation with interaction (FOCEI) and first-order conditional estimation with extended least square method (FOCE-ELS) with 19 (52.8%) studies, in addition to objective function value (OFV) with 17 (47.2%) studies and generalized additive model (GAM) with 1 study (7.7%). Almost all models reported internal evaluations, 35 (97.2%) studies, 25 (86.2%) of which reported bootstrap simulations and the methods generally used were goodness-of-fit plots model (GOF), visual predictive checks (VPC), prediction- and variability-corrected VPC (pvcVPC) and numerical predictive check (NPC); only 4 studies reported external evaluations. ^19^^,^^21^^,^^47^^,^^50^ A summary of demographics and PopPK modeling methods for all the included studies is presented in Table 2.

**Table 2.** Summary of population group characteristics and modeling methods by year.

	Year	Study	Country	Study design	Population	Sample size, (male/female)	Age (years), mean (SD) (range)	TBW (kg), mean (SD) (range)	eGFR (mL/min), mean CL _**CR**_ (SD)(range)	Compartments	Software	Analysis	Evaluation
** GENERAL**	1998	Yasuhara et al. ^26^	Japan	Retrospective	Patients infected with MRSA	190 (131/59)	64.3 (13.8)	52.3 (9.6)	77.1 (50.9)	Two compartments	NONMEM® version IV	NLME FOCE	Internal: GOF
	2009	Yamamoto et al. ^25^	Japan	Retrospective	Adult patients with a suspected or documented infection caused by gram positive bacteria and healthy subjects	100 (64/36) 6 (6/0)	65.4 (25.8-99.7) 21.7 (20-25)	52.6 (28.7-97) 60.3 (55.2-64.2)	89.3 (10.4)	Two compartments	NONMEM® version 5.1	NLME FOCE	Internal: Bootstrap
	2010	Tanaka et al. ^23^	Japan	Prospective	Patients infected with MRSA	164 (104/60)	74 (17-95)	53 (10)	65 (14-261)	One compartment	NONMEM® version 5	NLME	Internal
	2012	Purwonugroho et al. ^22^	Thailand	Prospective	Adult patients	212 (112/100)	66.62 (18.38)	57.64 (11.62)	35.07 (29.83)	Two compartments	NONMEM® version 7	NLME	Internal
	2013	Chung et al. ^17^	South Korea	Prospective	Adult patients with normal serum creatinine	678 (400/278)	56 (18-96)	62.3 (27-140)	NR	One compartment	NONMEM® version 7.1	NLME OFV	Internal: Bootstrap (n=2000), GOF, NPC
	2013	Deng et al. ^18^	China	Retrospective	Adult patients	72 (19/53)	54.07 (18.36)	61.12 (10.70)	82.09 (36.19)	One compartment	NONMEM® version 7.2	NLME	Internal: Bootstrap (n=2000), VPC
	2014	Lim et al. ^20^	South Korea	Prospective	Patients infected with MRSA	20 (15/5)	59.3 (12.9)	63.1 (15.7)	96.6 (31.1)	Two compartments	NONMEM®	NLME FOCE	Internal
	2018	Ji et al. ^19^	China	Retrospective	Patients who received continuous infusion of vancomycin and were not on renal replacement therapy	160 (106/54)	78 (42-95)	65 (38-90)	70.667 (42.74)	One compartment	NONMEM® version 7.3	NLME FOCEI OFV	Internal: Bootstrap (n=1000), NPDE; External (n= 58)
	2018	Usman et al. ^24^	Germany	Retrospective	Adult patients	144 (93/51)	62 (16-88)	79.5 (40-177)	89.8 (11.3-313.6)	One compartment	NONMEM® version 7.2	NLME FOCEI	Internal: Bootstrap (n=1000)
	2019	Liu et al. ^21^	China	Prospective	Adult patients	200 (128/72)	47.4 (15.42)	61.3 (12.06)	123.75 (59.96)	One compartment	NONMEM® version 7.3	NLME	Internal: Bootstrap (n=1000), GOF, VPC; External (n=74)
	2019	Bae et al. ^16^	South Korea	Retrospective	Adult patients	220 (139/81)	63 (21-98)	61.6 (30-126.7)	77.0 (4.57-279)	Two compartments	NONMEM® version 7.4	NLME FOCE	Internal: Bootstrap (n=1000), pvcVPC
	2020	Alqahtani et al. ^15^	Saudi Arabia	Retrospective	Adult patients older than 18 years old with cancer and non-cancer	74 (44/30)	55.1 (15.9)	75.5 (19.7)	102 (58.8)	One compartment	Monolix® version 4.4	SAEM	Internal: GOF, pvcVPC
**SURGICAL**	2016	Kim et al. ^29^	South Korea	Retrospective	Neurosurgical and non-neurosurgical patients	30 (14/16) 37 (20/17)	50.6 (15.0) 61.6 (15.7)	63.2 (11.6) 61.0 (12.7)	113.6 (48.3) 79.0 (44)	One compartment	NONMEM®	NLME FOCE OFV	Internal: Bootstrap (n=2000), VPC
	2018	Alqahtani et al. ^27^	Saudi Arabia	Prospective	Patients who underwent cardiac surgical	28 (17/11)	51.7 (15.9)	79.6 (17)	83.5 (29.3)	Two compartments	Monolix® version 4.4	SAEM	Internal: GOF, VPC
	2020	Jing et al. ^28^	China	Retrospective	Patients from the neurosurgery department, aged ≥18 years, receiving vancomycin therapy for ≥72 hours	222 (96/126)	46.95 (12.71)	60.22 (11.77)	115.8 (44.64)	One compartment	NONMEM® version 7.4.3	NLME FOCEI OFV	Internal: Bootstrap (n=2000), GOF, NPDE
	2021	Munir et al. ^30^	Pakistan	Prospective	Patients admitted to the surgical unit	58 (39/19)	54 (25-86)	75 (53-129)	101.15 (15.9-177.2)	One compartment	NONMEM® version 7.4.4	NLME FOCEI OFV	Internal: Bootstrap (n=1000), GOF, VPC
	2022	Wei et al. ^31^	China	Retrospective	Postoperative neurosurgical patients	560 (370/190)	52.41 (15.11)	69.74 (13.05)	112.74 (30.91)	One compartment	Phoenix NLME® version 8.3	NLME FOCE-ELS	Internal: Bootstrap (n=5000), GOF, VPC
**IMPAIRMENT KIDNEY FUNCTION**	1998	Schaedeli et al. ^37^	Switzerland	Retrospective	Patients undergoing long term hemodialysis who received vancomycin for infection therapy or prophylaxis	26 (16/10)	62 (15.2)	64.7 (13.6)	4.5 (4.3)	Two compartments	NONMEM®	NLME FOCE	Internal
	2018	Zaric et al. ^38^	Serbia	Retrospective	Patients with normal renal function and with mild to moderate chronic renal failure	32 (21/11) 78 (46/32)	59.15 (14.46) 67.00 (10.74)	81.37 (10.11) 78.52 (16.64)	54.38 (17.70)	Two compartments	NONMEM® version 7.3	NLME FOCE	Internal: Bootstrap (n=200), GOF
	2019	Kim DJ et al. ^33^	South Korea	Retrospective	Patients with vancomycin treatment for various infections, and at least two serum concentration measurements	99 (59/40)	64.8 (12.6)	59.7 (10.98)	54.49 (36.25)	Two compartments	NONMEM® version 7.4	NLME OFV	Internal: Bootstrap (n=1000), GOF
	2020	Ma et al. ^34^	China	Retrospective	Patients who received vancomycin as prophylactic medication following kidney transplant operation	56 (35/21)	43.72 (9.92)	58.27 (8.47)	41.95 (25.46)	One compartment	NONMEM® version 7.4	NLME FOCE OFV	Internal: GOF
	2020	Pai and DeBacker ^36^	USA	Retrospective	Patients with stable and unstable kidney disease	2640 (1689/950)	59 (16)	93.9 (28.1)	63 (39)	One compartment	Monolix® 2019R2	SAEM	Internal: Bootstrap (n=1000), NPDE
	2023	Oda et al. ^35^	Japan	Retrospective Prospective	Patients (age ≥ 18 years) who had received intermittent hemodialysis therapy for end-stage kidney disease	28 (8/20)	61 (14.5)	57.8 (13.2)	9.6 (4.7)	Two compartments	NONMEM® version 7.3; R version 4.1.2	NLME OFV	Internal: Bootstrap, GOF, pvcVPC
	2024	Ahmed et al. ^32^	Sudan	Retrospective	Adult patients with renal impairment	99 (66/33)	65 (50-75)	NR	12.7 (5.52-25.78)	One compartment	Monolix® 2020R1	NLME SAEM	Internal: Bootstrap (n=1000), NPDE, GOF, VPC
**OBESE**	2015	Adane et al. ^39^	USA	Prospective	Extremely obese adult patients (BMI ≥ 40 kg/m2) with suspected or confirmed Staphylococcus aureus infection	31 (19/12)	43 (38.5-53)	147.6 (142.8-178.3)	124.8 (106.0-133.9)	Two compartments	NONMEM® version 7.3	NLME FOCE OFV	Internal
	2018	Crass et al. ^40^	USA	Retrospective	Obese (BMI ≥ 30 kg/m2) adult patients aged 18–90 years who underwent peak and trough vancomycin	346 (183/163)	57 (14)	132.5 (32.6)	171 (75)	One compartment	R environment Pmetrics ^TM^ package	NPAG	NR
	2024	Polásková et al. ^41^	Czech Republic	Retrospective	Obese adult patients (age ≥ 18years; BMI ≥ 30kg/m ^2^) treated with intravenous vancomycin during intermittent hemodialysis	27 (14/13)	69 (58-72)	102 (91.5-118)	NR	One compartment	Monolix® 2021R2	NLME SAEM OFV	Internal: Bootstrap (n=1000), NPDE, GOF
**GERIATRICS**	2010	Sanchez et al. ^43^	USA	Retrospective	Adult and geriatric patients	141 (NR)	55 (14.58)	73.2 (17.48)	NR	Two compartments	NONMEM® version VI	NLME	Internal: Bootstrap (n=200)
	2019	Zhou et al. ^45^	China	Retrospective	Elderly patients (age ≥ 65 years) with HAP or CAP	70 (49/21)	78.3 (6.96)	60.7 (10.2)	56.3 (22.1)	One compartment	NONMEM® version 7.3	NLME FOCEI OFV	Internal: Bootstrap (n=1000), NPDE, GOF
	2020	Zhang et al. ^44^	China	Prospective	Elderly patients (age ≥ 65 years) infected	150 (104/46)	73.6 (6.83)	61.7 (1 1.1)	84.1 (25.6)	One compartment	NONMEM® version 7.4	NLME FOCEI OFV	Internal: Bootstrap (n=2000), NPDE
	2024	Ling et al. ^42^	China	Retrospective	Inpatients with a diagnosis of MRSA or suspected of having a positive drug resistant bacteria infection	313 (201/112)	72 (65-95)	65 (38-110)	70.98 (16.75-165.39)	One compartment	NONMEM® version 7.3 R version 2.15.1	NLME OFV	Internal: Bootstrap (n=2000), NPDE, GOF
**CANCER**	2005	Buelga et al. ^46^	Spain	Retrospective	Adult (15-year-old) in patients with an underlying hematological malignancy admitted for suspected or documented infection caused by gram-positive bacteria	215 (119/96)	51.5 (15.9)	64.7 (11.3)	89.4 (39.2)	One compartment	NONMEM® version 5.1.1	NLME OFV GAM	Internal
	2018	Okada et al. ^47^	Japan	Retrospective	Patients undergoing allo-HSCT who received preventive treatment with vancomycin	75 (49/26)	49 (17-69)	59.4 (39.4-104.5)	113 (47-253)	Two compartments	Phoenix NLME® version 7	NLME FOCE-ELS OFV	Internal: Bootstrap (n=1000), GOF, VPC; external: (20 patients)
	2020	Alqahtani et al. ^15^	Saudi Arabia	Retrospective	Adult patients older than 18 years old with cancer and non-cancer.	73 (58/42)	53.8 (15.7)	72.7 (16.2)	102 (58.8)	One compartment	Monolix® version 4.4	SAEM OFV	Internal: GOF, pvcVPC
	2023	Tsuda et al. ^48^	Japan	Retrospective	Patients with solid or hematological malignancy	325 (182/143)	67.8 (14.8)	54 (12)	80 (46.7)	One compartment	NONMEM® version 7.4.3	NLME FOCEI OFV	Internal: Bootstrap (n=1000), GOF, pvcVPC
**CYSTIC FIBROSIS**	2024	Yellepeddi et al. ^49^	USA	Retrospective	Adults with cystic fibrosis	19 (5/14)	31.2 (12.5)	63.6 (17.1)	106.6 (37.9)	One compartment	NONMEM® version 7.5	NLME FOCEI OFV	Internal: Bootstrap (n=1000), GOF, VPC
**TRAUMA**	2015	Medellín-Garibay et al. ^50^	Spain	Retrospective	Adult patients from the Traumatology service with proven or suspected infection	118 (53/65)	74.3 (14)	72.0 (15)	90.5 (51.67)	Two compartments	NONMEM® version 7.2	NLME	Internal: Bootstrap (n=200); External, (n=40)

**CL**_**CR**_ = Creatinine clearance calculated by Cockcroft-Gault formula; **eGFR** = estimated glomerular filtration rate; **FOCE** = first-order conditional estimation; **FOCEI** = First-order conditional estimation with interaction; **FOCE-ELS** = first-order conditional estimation with extended least square method; **GAM** = generalized additive model; **GOF** = goodness-of-fit plots model; **MRSA** = methicillin resistant Staphylococcus aureus; **NLME** = Nonlinear mixed-effects modeling; **NPAG** = nonparametric adaptive grid; **NPC** = numerical predictive check; **NPDE** = normalized prediction distribution errors; **NR** = no reported; **OFV** = objective function value; **pvcVPC** = prediction- and variability-corrected VPC; **SAEM** = stochastic approximation expectation maximization; **SD** = standard deviation; **USA** = United States of America; **VPC** = visual predictive checks.

The combined mean and combined SD of age did not differ much from the combined means and SD by groups, being for all 59.74 years and 17.24 years respectively; as for the total body weight (TBW) the obese group presented a combined mean of 131.62 kg with a combined SD of 31.59 kg, while the total of the groups had a combined mean of 76.98 kg and a combined SD of 21.25 kg; for the estimated glomerular filtration rate (eGFR) greater heterogeneity was found, since in the group with impaired kidney function the combined means and SD were 59.55 ml/min and 44.54 ml/min respectively, while in the total the combined mean of the eGFR was 80.42 ml/min and the combined SD was 54.07 ml/min (see Extended data E2 Additional results tables).

Most of the equations presented by the PopPK models are in the form in which the expressions for clearance (CL), distribution volumes (V _i_), intercompartmental clearance (Q) and elimination transfer rate constants (k _12_, k _21_), are equal to the estimates of the population mean of each study (CLpop, V _i_pop, Qpop, k _12_pop, k _21_pop) or typical values (TVCL, TVV _i_, TVQ, TVk _12_, TVk _21_), which as in the case of CL are generally affected proportionally or additively by covariates, in greater proportion by the renal clearance (CL _CR_) or the estimated glomerular filtration rate (eGFR) by Cockroft-Gault, although the studies by Chung et al. ^17^ and Ling et al. ^42^ uses cystatin C to affect the TVCL or the study by Medellín-Garibay ^50^ and Wei et al. ^31^ they associate furosemide or mannitol respectively as factors that alter eGFR, for these models serum creatinine or CLCR are also included as covariates. Furosemide was not directly used to estimate the glomerular filtration rate (GFR). Instead, it was administered as part of a furosemide stress test, which has been proposed as a functional marker of renal reserve. This test assesses renal response to a standardized dose of furosemide and has been used to predict acute kidney injury (AKI) progression in critically ill patients. However, in the context of the referenced study, cystatin C was the primary biomarker used for GFR estimation.

To a lesser extent, total body weight (TBW) or age are reported as covariates for TVCL, other models include clinical conditions such as the use of hemodialysis (HD), ^16^^,^^35^ continuous renal replacement therapy (CRRT) ^16^ or intermittent renal replacement therapy (IRRT) ^37^; there are models such as that of Kim et al. ^29^ that includes as covariates being a neurosurgical patient, presenting underlying liver cirrhosis or co-administration of nephrotoxic drugs; the most recent model such as that of Tsuda et al. ^48^ even includes quick SOFA (qSOFA) as a covariate. Other covariates only presented once per model, such as sex, ^40^ daily dose of vancomycin and AST, ^38^ albumin ^27^ and post-craniotomy meningitis. ^44^

Regarding distribution volumes, they are most commonly reported as equal to TVVi if expressed in liters (L) or as the relative TVVi by TBW if expressed in L/kg. In some equations, age can also influence these values. The most reported equation patterns for CL, V _i_, Q, k12, and k21 are:

$$\mathrm{CL}=\text{TVCL}\times {\left({\mathrm{CL}}_{\mathrm{CR}}/{\bar{\mathrm{CL}}}_{\mathrm{CR}}\right)}^{\theta_{{\mathrm{CL}}_{\mathrm{CR}}}}$$

$$\mathrm{CL}=\theta_{{\mathrm{CL}}_{\mathrm{CR}}}\times {\mathrm{CL}}_{\mathrm{CR}}$$

CL=TVCL

$$\mathrm{CL}=\text{TVCL}+\left(\theta_{{\mathrm{CL}}_{\mathrm{CR}}}\times {\mathrm{CL}}_{\mathrm{CR}}\right)$$

$$\mathrm{CL}=\text{TVCL}\times {\left(\mathrm{TBW}/\bar{\mathrm{TBW}}\right)}^{\theta_{\mathrm{TBW}}}$$

$$\mathrm{CL}=\text{TVCL}\times \left({\mathrm{CL}}_{\mathrm{CR}}/{\bar{\mathrm{CL}}}_{\mathrm{CR}}\right)$$

$$\mathrm{CL}=\text{TVCL}\times {\left({\mathrm{CL}}_{\mathrm{CR}}/{\bar{\mathrm{CL}}}_{\mathrm{CR}}\right)}^{\theta_{{\mathrm{CL}}_{\mathrm{CR}}}}\times {\left[\mathrm{TBW}/\bar{\mathrm{TBW}}\right]}^{\theta_{\mathrm{TBW}}}$$

$$V_{i}=\mathrm{TVV}$$

$$V_{i}=\text{TVV}\times \text{TBW}$$

Q=TVQ

$$k_{12}={\mathrm{TVk}}_{12}$$

$$k_{21}={\mathrm{TVk}}_{21}$$

The main features and values of the equations, parameters, population mean (VT) and variability are shown in Table 3. Many of the studies do not explicitly show TV for which we calculate with measures of central tendency for the reported covariates and substituting them in the covariate equations in the final model; although most studies with two-compartment models reported parameters in the form of flow rates (CL and Q), two studies reported model parameters in the form of elimination, transfer rate constants (k _12_, k _21_) were presented, in order to make comparisons among studies, the conversion of parameters in the form of flow rates was implemented with the following equation:

$$Q=k_{12}\times V_{1}$$

**Table 3.** Main features of the published PopPk models.

	Study				Volume of distribution related expressions: V _**i**_ (L)			Population mean (TV)		BSV (ω)		RV ( *a*),( *b*)	
		Equations	Parameter	Value	Equations	Parameter	Value	CL (L/h), Q (L/h), k _**ij**_ (h ^**-1**^)	V _**i**_ (L)	CL	V _**i**_	Additive (mg/L)	Proportional
** GENERAL**	Yasuhara et al. ^26^	CL _CR_ ≤ 85 mL/min: CL = θ _1_ × CL _CR_ CL _CR_ > 85 mL/min: CL = θ _2_ k _12_ = θ _3_ k _21_ = θ _4_	θ _1_ θ _2_ θ _3_ θ _4_	0.0478 3.51 0.525 0.213	V _ss_ = θ _5_	θ _5_	60,7	3.51	60.7	38.5%	V _ss_ = 25.4%	NR	23.7%
	Yamamoto et al. ^25^	CL _CR_ > 85 mL/min: CL = θ _1_ CL _CR_ ≤ 85 mL/min: CL = θ _2_ × CL _CR_ + θ _3_ Q = θ _8_	θ _1_ θ _2_ θ _3_ θ _8_	3.83 0.0322 0.32 8.81	V _1_ = θ _4_ × (1 + (θ _5_ × STATUS)) × TBW V _2_ = θ _6_ + (STATUS × θ _7_)	θ _4_ θ _5_ θ _6_ θ _7_	0.206 0.272 39.4 21.2	CL = 3.83 Q = 8.81	V _1_ = 28.82 V _2_ = 60.6	37.5%	V _1_ = 18.2% V _2_ = 72.8%	NR	14.3%
	Tanaka et al. ^23^	CL (ml/min) = θ _1_ × eGFR	θ _1_	0.875	V (L/kg) = θ _2_	θ _2_	0.864	2.68	45.79	19.8%	30.7%	12.7	NR
	Purwonugroho et al. ^22^	CL = θ _1_ × CL _CR_ (mL/min) Q = θ _3_	θ _1_ θ _3_	0.044 6.950	V _1_ = θ _2_ × Age V _2_ = θ _4_	θ _2_ θ _4_	0.542 44.2	CL = 1.56 Q = 6.95	V _1_ = 36.11 V _2_ = 44.2	35.78%	V _1_ = 20.93% V _2_ = 57.27%	4.51	NR
	Chung et al. ^17^	CL = 4.9 × (1 + θ _1_ × [AGE-57]) × (1 + θ _2_ × [TBW - 60.8]) × (1 + θ _3_ × [SCr - 0.8]) × (CystatinC/0.91)^θ _4_ if female, apply 1 + θ _5_	θ _1_ θ _2_ θ _3_ θ _4_ θ _5_	-0.0042 0.00997 -0.322 -0.780 -0.150	V = 46.2× (1 + θ _6_ × [AGE-57]) × (1+ θ _7_ × [TBW-60.8]) if female, apply 1 + θ _8_	θ _6_ θ _7_ θ _8_	0.00580 0.00661 -0.119	4.90	46.2	26.2%	37.3%	1.40	6.39%
	Deng et al. ^18^	CLCR < 80 mL/min: CL = θ _1_ × CL _CR_ CLCR ≥ 80 mL/min: CL = θ _2_	θ _1_ θ _2_	0.0654 4.9	V = θ _3_	θ _3_	47.76	4.90	47.76	45.35 %	39.25 %	1.21	30.71%
	Lim et al. ^20^	CL = θ _1_ × CL _CR_/100 Q = θ _4_	θ _1_ θ _4_	3.96 6.99	V _1_ = θ _2_ V _2_ = θ _3_	θ _2_ θ _3_	33.1 48.3	CL = 3.96 Q = 6.99	V _1_ = 33.1 V _2_ = 48.3	40.1%	35.7%	NR	0.231 (SD)
	Medellín-Garibay et al. ^50^	Furosemide = 0: CL = θ _1_ × CL _CR_ Furosemide = 1: CL = θ _5_ × CL _CR_ Q = θ _3_	θ _1_ θ _5_ θ _3_	0.49 0.34 0.81	If age > 65 years: V _1_ (L/kg) = θ _2_ × TBW V _2_ (L/kg) = θ _4_ × TBW If age ≤ 65 years: V _1_ (L/kg) = θ _6_ × TBW	θ _2_ θ _4_ θ _6_	1.07 5.99 0.74	CL = 2.6 (1.85) Q = 0.81	V _1_ = 77.4 (53.28) V _2_ = 424.8	36.2%	V _1_ = 37.1% V _2_ = NR	NR	19.3%
	Ji et al. ^19^	CL = θ _1_ × (1 + θ _2_ × [CL _CR_ - 80]) × (75/AGE)^θ _3_	θ _1_ θ _2_ θ _3_	2.829 0.00842 0.8143	V = θ _4_	θ _4_	52.14	2.829	52.14	32.42%	28.87%	2.64	26.79%
	Usman et al. ^24^	CL = θ _1_ × (1 + θ _2_ × [CL _CR_ − θ _3_])	θ _1_ θ _2_ θ _3_	2.32 0.0018 89.8	V = θ _4_	θ _4_	19.2	2.32	19.2	20.40%	NR	NR	38.50%
	Liu et al. ^21^	CL = θ _1_ × (eGFR/105.5)^θ _2_ × (AGE/48.5)^θ _3_ × (TBW/60)^θ _4_	θ _1_ θ _2_ θ _3_ θ _4_	5.07 0.524 -0.309 0.491	V = θ _5_	θ _5_	46.3	5.07	46.3	20.80%	18.10%	1.28	15.90%
	Bae et al. ^16^	CL = θ _1_ × (CL _CR_/72)^θ _2_ CL _CRRT_ = θ _3_ CL _HD_ = θ _4_ Q = θ _6_	θ _1_ θ _2_ θ _3_ θ _6_	2.82 0.836 0.716 0.334 11.7	V _1_ = θ _4_ V _2_ = θ _5_ × (TBW/60)	θ _4_ θ _5_	31.8 75.4	CL = 2.80 Q = 11.7	V _1_ = 31.8 V _2_ = 75.4	99.20%	V _1_ = NR V _2_ = 49.20%	NR	0.253 (SD)
	Alqahtani et al. ^15^	CL = θ _1_ × (CL _CR_/96.3)^θ _2_	θ _1_ θ _2_	5.6 0.18	V = θ _3_	θ _3_	42	5.6	42	20.3%	18.2%	NR	23%
**SURGICAL**	Kim et al. ^29^	CL = [early phase θ _1_ or late phase θ _2_] × (CL _CR_/95.8) × θ _3_^TO×I^ × θ _4_^LC^ + θ _5_^NEUR^	θ _1_ θ _2_ θ _3_ θ _4_ θ _5_	4.36 3.69 0.811 0.511 2.42	V = [early phase θ _6_ or late phase θ _7_]	θ _6_ θ _7_	83.7 (107)	4.36 (3.69)	83.7 (107)	0.125 variance	NR	1.92	8.59%
	Alqahtani et al. ^27^	CL = θ _1_ × (CL _CR_/83.5)^0.514 × (ALBUMIN/35.5)^0.854 Q = θ _2_	θ _1_ θ _2_	6.13 0.22	V _1_ = θ _3_ × (TBW/79.6)^0.466 V _2_ = θ _4_	θ _3_ θ _4_	40 3.88	CL = 6.13 Q = 0.22	V _1_ = 40 V _2_ = 3.88	22.1%	V _1_ = 6.34% V _2_ = 61.2%	0.055	15.2%
	Jing et al. ^28^	CL = [6.4 × (eGFR/128)^θ _1_ × (TBW/60) (AGE/47)^θ _3_] × e×p^θ _4_	θ _1_ θ _2_ θ _3_ θ _4_	0.515 0.417 0.267 0.0417	V = θ _4_	θ _4_	60.1	6.49	60.2	7%	NR	NR	9%
	Munir et al. ^30^	By CL _CR_: CL = 1 + θ _1_ × (CL _CR_ − 101.15) By TBW: CL = 1 − θ _2_ × (TBW − 75)	θ _1_ θ _2_	0.0046 0.011	V = θ _3_	θ _3_	22.6	2.45	22.6	11.3%	22.8%	3.07	NR
	Wei et al. ^31^	CL = 7.98 × (eGFR/115.2)^θ _1_ × (TBW/70)^θ _2_ × e×p^A with mannitol, A = 0.13; otherwise, A = 0	θ _1_ θ _2_	0.8 0.3	V = θ _3_	θ _3_	60.2	7.98	60.2	48.19%	NR	2.73	13.06%
**RENAL**	Schaedeli et al. ^37^	CL _CR_ ≥ 2 mL/min: CL = θ _1_+ θ _2_ × CL _CR_ CL _CR_ < 2 mL/min: CL = θ _1_ CLDv = θ _3_ × CLD _BUN_ k _12_ = θ _5_ k _21_ = θ _6_	θ _1_ θ _2_ θ _3_ θ _5_ θ _6_	2.25 0.585 0.336 0.872 0.162	V _c_ = θ _4_ × TBW V _ss_ = θ _5_ × TBW	θ _4_ θ _5_	0.164 1.05	CL = 2.25 k _12_ = 0.872 k _21_ = 0.162	V _ss_ = 67.93	CLCR < 2 mL/min: Cl = 90% CLCR ≥ 2 mL/min: Cl = 32%	22%	NR	13%
	Zaric et al. ^38^	Normal renal function: CL = θ _1_ + θ _3_ × FIB Impaired renal function: CL = θ _2_ + θ _4_ × DD + 0.00194 × AST	θ _1_ θ _2_ θ _3_ θ _4_ θ _5_	0.0727 0.284 0.205 0.000596 0.00194	Normal renal function: V _1_ = θ _6_ Impaired renal function: V _1_ = θ _7_	θ _6_ θ _7_	7.47 29.9	0.284	29.9	0.059 variance 0.135 variance	NR	0.05 variance 0.045 variance	NR
	Kim DJ et al. ^33^	CL = θ _1_ × [(θ _2_/ eGFR _base_) + (eGFR _at time_/eGFR _median_)] Q = θ _5_	θ _1_ θ _2_ θ _5_	2.21 0.921 3.06	V _1_ = θ _3_ V _2_ = θ _4_	θ _3_ θ _4_	32.6 45.8	CL = 2.21 Q = 3.06	V _1_ = 32.6 V _2_ = 45.8	5.3%	V _1_ = NR V _2_ = 32%	1.95	14.3%
	Ma et al. ^34^	CL = θ _1_ × [(TBW/59.95)^θ _2_] × [(eGFR/36.67)^θ _3_]	θ _1_ θ _2_ θ _3_	2.08 0.698 1.07	V = θ _4_ × [(TBW/59.95)^θ _5_]	θ _4_ θ _5_	63.2 0.934	2.08	63.2	21.5%	NR	NR	24.2%
	Pai and DeBacker ^36^	CL = e×p(θ _1_ + θ _2_ × (eGFR/100)) - θ _3_	θ _1_ θ _2_ θ _3_	1.03 0.737 -1.63	V = θ _4_	θ _4_	66.4	0.334	66.4	(0.44, 0.85) IQR	(60.5, 98.2) IQR	0.76	NR
	Oda et al. ^35^	CL = θ _1_ × (TBW/70)^0.75 × e×p (η _CL_) + unbound fraction × KoA-predicted CL _HD_ if (during HD) 1 else 0 k _12_ = θ _3_ k _21_ = θ _4_ × e×p (η _k21_) η _(CL,K21)_ is a random variable number depending on the mean of zero with a variance of ω ^2^_(CL,k21)_	θ _1_ θ _3_ θ _4_	0.316 0.525 0.213	V _SS_ = θ _2_ × TBW × e×p (η _Vss_) η _Vss_ is a random variable number depending on the mean of zero with a variance of ω ^2^_Vss_	θ _2_	1.160	CL = 0.316 k _12_ = 0.525 k _21_ = 0.213	V _SS_ = 67.05	0.365 variance	0.302 variance	0.064 variance	NR
	Ahmed et al. ^32^	CL = θ _1_ × TZ _R_^θ _2_ × e×p (η _CL_) η _CL_ is a random variable number depending on the mean of zero with a variance of ω ^2^_CL_	θ _1_ θ _2_	2.02 40.49	V = θ _2_ × e×p (η _V_) η _V_ is a random variable number depending on the mean of zero with a variance of ω ^2^_V_	θ _2_	65	2.02	65	0.46 (SD)	0.39 (SD)	NR	0.28 (SD)
**OBESE**	Adane et al. ^39^	CL = θ _2_ × (CL _CR_/125)	θ _2_	6.54	V = θ _1_ × TBW	θ _1_	0.51	6.54	75.43	26.70 %	23.90 %	NR	18.9%
	Crass et al. ^40^	CL = θ _1_ - θ _2_ × AGE - θ _3_ × (SCr)+ θ _4_ × SEX + θ _5_ × TBW ^0.75^	θ _1_ θ _2_ θ _3_ θ _4_ θ _5_	8.688 0.075 1.988 1.245 0.073	V = θ _6_	θ _6_	73.969	5.9	74.1	39.94%	33.20%	NR	NR
	Polásková et al. ^41^	CL = θ _1_	θ _1_	0.83	V = θ _2_ × e×p^(θ _3_ × LBM)	θ _2_ θ _3_	26.39 0.015	0.83	26.39	0.39 (SD)	0.39 (SD)	NR	0.13 (SD)
**GERIATRICS**	Sanchez et al. ^43^	CL = θ _1_+θ _5_ × CL _CR_ Q = θ _4_ × TBW	θ _1_ θ _5_ θ _4_	0.157 0.563 0.111	V _1_ = θ _2_ × TBW V _2_ = θ _3_ × AGE/53.5	θ _2_ θ _3_	0.283 32.2	CL = 2.21 Q = 8.12	V _1_ = 20.71 V _2_ = 44.5	24.49 %	V _1_ = NR V _2_ = 6.8 %	NR	24.9%
	Zhou et al. ^45^	CL = θ _1_ × (CL _CR_/56.28)^θ _2_	θ _1_ θ _2_	2.45 0.542	V = θ _3_	θ _3_	154	2.45	154	17.53%	34.90%	NR	6.57%
	Zhang et al. ^44^	CL = θ _1_ × (eGFR/80)^θ _2_ × (1 + θ _3_ × PCM)	θ _1_ θ _2_ θ _3_	3.74 1.03 0.41	V = θ _4_	θ _4_	118	3.74	118	44.26%	54.99%	0.184 (log scale)	NR
	Ling et al. ^42^	eGFR by CKD-EPIcys-scr: CL = 3.79 × (eGFR/ 64.82)^θ _1_ × (TBW/65)^θ _3_ eGFR by BIS-2: CL = 3.71 × (eGFR/ 59.53)^θ _2_ × (TBW/65)^θ _3_	θ _1_ θ _2_ θ _3_	1.06 1.11 0.575	V = θ _4_	θ _4_	76.9	3.79	76.9	23.6%	NR	0.7	23.2%
**CANCER**	Buelga et al. ^46^	CL = θ _1_ × CL _CR_	θ _1_	1.08	V = θ _2_ × TBW	θ _2_	0.98	5.79	63.40	28.16%	37.15%	3.52	NR
	Okada et al. ^47^	CL = θ _2_ × (CL _CR_/113)^θ _6_ Q = θ _4_	θ _2_ θ _6_ θ _4_	4.25 0.70 1.95	V _1_ = θ _1_ × (TBW/59.4)^θ _5_ V _2_ = θ _3_	θ _1_ θ _5_ θ _3_	39.2 0.78 56.1	CL = 4.25 Q = 1.95	V _1_ = 39.2 V _2_ = 56.1	25.2%	V _1_ = 14.2% V _2_ = 66.9%	NR	17.2%
	Alqahtani et al. ^15^	CL = θ _1_ × (CL _CR_/99.9)^θ _2_	θ _1_ θ _2_	7.4 0.21	V = θ _3_	θ _3_	45	7.4	45	15.9%	13.8%	NR	12.5%
	Tsuda et al. ^48^	CL = θ _1_ × (CL _CR_ ∕ 4.2)^θ _2_ × ƒqSOFA ƒqSOFA is 1 when qSOFA scores of 0 and it is 0 when qSOFA scores are 1 or greater	θ _1_ θ _2_	2.8 0.8	V = 0.17 × AGE + 0.22 × TBW + 15	NR	NR	2.8	38.40	28%	NR	NR	23.2%
**CYSTIC FIBROSIS**	Yellepeddi et al. ^49^	CL = θ _1_ × (TBW/52.6)^θ _2_	θ _1_ θ _2_	5.52 0.5	V = θ _3_	θ _3_	31.5	5.52	31.5	23%	NR	NR	0.0413 variance
**TRAUMA**	Medellín-Garibay et al. ^50^	Furosemide = 0: CL = θ _1_ × CL _CR_ Furosemide = 1: CL = θ _5_ × CL _CR_ Q = θ _3_	θ _1_ θ _5_ θ _3_	0.49 0.34 0.81	If age > 65 years: V _1_ (L/kg) = θ _2_ × TBW V _2_ (L/kg) = θ _4_ × TBW If age ≤ 65 years: V _1_ (L/kg) = θ _6_ × TBW	θ _2_ θ _4_ θ _6_	1.07 5.99 0.74	CL = 2.6 (1.85) Q = 0.81	V _1_ = 77.4 (53.28) V _2_ = 424.8	36.2%	V _1_ = 37.1% V _2_ = NR	NR	19.3%

**AST** = aspartate aminotransferase (IU/L); **BIS-2** = Berlin Initiative Study 2; **BSV** = between-subject variability; **BUN** = blood urea nitrogen; **CKD-EPIcys-scr** = Chronic Kidney Disease Epidemiology Collaboration for cystatin C and creatinine; **CL** = Clearance; **CL**_**CR**_ = creatinine clearance calculated by Cockcroft-Gault formula; **CL**_**HD**_ = hemodialysis clearance; **CL**_**CRRT**_ = CL in patients with continuous renal replacement therapy; CL in patients with hemodialysis; **CLD**_**BUN**_ = in vivo urea dyalisis clearance; **CLDv** = vancomycin dialysis filter clearance; **DD** = daily dose (mg/day); **eGFR** = estimated glomerular filtration rate; **FIB** = Fibrinogen; **HD** = hemodialysis; **IQR** = Interquartile Range; **KoA** = dialyzer mass transfer-area coefficient; **LC** = underlying liver cirrhosis; **LBM** = lean body mass; **NR** = no reported; **NEUR** = neurosurgical patient; **PCM** = post-craniotomy meningitis; **RV** = residual variability; **SD** = standard deviation; **SCr** = serum creatinine; **STATUS** = Value 1 for patients with gram-positive infections; **TBW** = total body weight; **TOXI** = co-administration of nephrotoxic drug; **TV** = typical value; **Vc** = central volume; **Vd** = volume of distribution; **Vp** = plasma volume; **Vss** = steady-state volume of distribution.

To perform an analysis of the TV, the combined means of all studies and also by compartments were calculated; the complete results are found in Supplementary data S2. The TVCL for all studies was 3.02 L/h; by groups the TVCL was 3.76 L/h for the general population, 7.08 L/h for surgical patients, 0.5 L/h for the group with impaired renal function, 5.61 L/h for obese patients, 3.31 L/h for geriatric patients, 4.38 L/h for patients with cancer, 5.52 L/h for cystic fibrosis and 2.6 L/h for trauma patients; when separating the patients without impaired renal function, the TVCL is 4.5 L/h, which differs substantially from that reported in the group with impaired renal function and shows the change with respect to the TVCL of all studies when eliminating those with the lowest clearance.

The TV of the central distribution volume (TVVc) for all studies was 58.24 L, by groups the TVVc was 42.74 L for the general population, 57.93 L surgical, 64.8 L for the group with impaired renal function, 71.01 L obese, 82.3 L geriatric, 47 L with cancer, 31.5 L with cystic fibrosis and 74.4 L for trauma patients; the TVVc without the obese, geriatric and trauma group is 54.78 L, while in the obese, geriatric and trauma group the TVVc was 78 L. The TVCL for single compartment models was 4.38 L/h and TVV was 61.26 L. The TVCL for two compartment models was 2.63 L/h, TVQ was 8.71 L/h, TVV1 and TVV2 were 38.59 L and 96.97 L respectively.

Regarding the variability of the TV, the mean between-subject variability coefficients of CL (ωCL) were 31.44% (max: 99.20%; min: 5.30%), of the central distribution volume (ωVc) 27.29% (max: 54.99; min: 6.34%) and peripheral (ωVp) 49.45% (max: 72.80%; min: 6.80%); and finally the means of additive ( *a*) and proportional ( *b*) errors were 6.67 mg/L (max: 55.00 mg/L; min: 6.34 mg/L) and 27.29% (max: 54.99; min: 0.70%) respectively. The previously mentioned results are summarized in Supplementary data S2.

## Discussion

In the precision dosing, the TDM and development of PopPK within the MIPD is relevant to improve efficacy and/or lower toxicity in special populations with high variability, like pediatrics, elderly, those with renal or hepatic impairment and comedicated patients. The translation of this approach personalized medicine requires the implementation of new dosing scenarios, new working paradigms and clinical pharmacology experts and researchers, that are not limited only to the academic area. ^51^

NONMEM (ICON, Dublin, Ireland), Monolix (Lixoft, Paris, France) and Phoenix NLME (Certara, Princeton, NJ) are the most widely used nonlinear mixed effects modeling (NLMEM) tools in pharmacometrics. They are commercial offerings with fees substantial licensing costs, and while all have programs aimed at reducing or eliminating licensing costs in educational institutions or low-income countries, the administrative hurdles and associated delays in availability can be cumbersome when conducting analysis and training students and researchers to use these tools in resource-limited settings. Implementation of open-source software based on R and the nlmixr package may be a credible and capable alternative to commercial PK/PD modeling software to fit compartmental of pharmacokinetic/pharmacodynamic (PK/PD) models described by ordinary differential equations (ODEs). Parameter estimation algorithms implemented in nlmixr currently include relatively mature implementations of NLMEM, SAEM, and first-order, first-order with interaction, FOCE, and FOCEI. ^52^

About the development of PopPK models we can observe for vancomycin it is expected that CL _CR_ would be the most important covariable in most models and could affect the CL and the prediction of serum vancomycin concentration, it is because vancomycin is excreted 80% to 90% as an unchanged drug in urine ^19^; conventionally, the eGFR is calculated by the Cockroft-Gault equation, however, the CKD-EPI (Chronic Kidney Disease Epidemiology Collaboration), Modification of Diet in Renal Disease (MDRD) and Berlin Initiative Study 2 (BIS-2) equations has been shown to be more accurate, especially in youngest. ^53^ This is because, although the Cockcroft-Gault equation is widely used in pharmacokinetic studies and drug dosing adjustments, it has several limitations. It was developed in a specific population, primarily adult males, which limits its applicability to other groups, such as elderly individuals, patients with altered body composition (e.g., obesity, cachexia), or critically ill patients. Additionally, since it relies on serum creatinine levels, it is influenced by factors such as muscle mass, diet, and hydration status, potentially leading to inaccurate estimations of renal function. The equation also lacks standardization across different creatinine assay methods, and its accuracy diminishes in patients with very low or highly fluctuating glomerular filtration rates (GFR), such as those with acute kidney injury (AKI). Moreover, the use of actual body weight can introduce further errors, particularly in patients with obesity or fluid overload. Despite these limitations, the Cockcroft-Gault equation remains widely used due to its historical application in drug dosing guidelines and its inclusion in many pharmacokinetic models.

Ling et al. used to covariate the model CKD-EPIcys and BIS-2 eGFR with specific equations for each one. ^42^ Beyond that, models have always been compared to the CL _CR_, including the studies like Tanaka et al. which uses cystatin C, those who consider that this may be more accurate and sensitive than creatinine for calculating eGFR, suggesting that it could be a good predictive marker of CL and vancomycin concentrations. ^23^

Great difference was found in TV estimates, the population with significantly higher TVCL and TVV are obese and surgical patients; in both, this finding in TVCL are explained by the augmented renal clearance (ARC) in early stage of the surgical approach or in obese by the compensatory vasodilation of the afferent arteriole, ^54^ also in neurological patients the brain lesions and the loss of autoregulation induced by brain injury may impair the kidney autoregulatory process ^29^; in the obese population because the volume of distribution is linked to weight and also to the constants of CL _CR_, it is expected that both the TVCL and the TVV increase. ^39^ The trauma and elderly have also the highest TVV (central and peripheral) but lowest TVCL; Variability in trauma patients CL is due to the fact that the elimination of vancomycin depending on tubular secretion and the concomitant administration of other drugs, such as furosemide ^50^; the renal function of the elderly gradually decreases with age and the larger volume of distribution may be by the changes in the peripheral circulation usually due to poor nutrition, hypoalbuminemia and internal environmental disorders such as hypokalemia, hyponatremia and metabolic acidosis that increased tissue affinity for vancomycin, and the TVV is high because they are attached to the weight. ^45^ In patients with impairment kidney function the heterogenicity of the TVCL it is due to changes in the central compartment generated by renal effect of the vancomycin, dialysis and changes in the ultrafiltration rate of each session, for this reason eGFR estimated by Cockroft-Gault equation is not a reliable marker of renal function. ^33^^,^^36^^,^^37^ Patients with above-the-mean vancomycin clearance and volume of distribution typically exhibit pharmacokinetic profiles associated with increased drug elimination and expanded drug distribution. Several factors may contribute to these elevated parameters, including younger age, preserved or augmented renal function, higher body weight, and conditions associated with hyperdynamic circulation, such as sepsis or burns. Higher clearance rates may result in subtherapeutic vancomycin concentrations, potentially reducing efficacy and increasing the risk of treatment failure, particularly in infections caused by less susceptible pathogens. Similarly, an increased volume of distribution may lead to lower peak concentrations, which could impact the drug’s time-dependent antibacterial activity. Given these considerations, patients with above-the-mean clearance and volume of distribution may require individualized dosing strategies, such as higher initial doses, more frequent administration, or therapeutic drug monitoring to ensure optimal target attainment while minimizing the risk of underexposure. ^55^^–^^57^

To end when we look at the variability of the models is striking that for the patients undergoing allogeneic transplantation, the models developed indicate a high variability due to high between-subject variability and the difficulty of maintaining the therapeutic range, due to the characteristics of these patients with extremely low hematocrit levels, increased intravascular volume, and increased renal clearance. ^46^^–^^48^ It is important to note that this review had several limitations. Some of the papers do not specify the clinical and pathological characteristics of the study subjects. The creatinine clearance formulas are different in every article making necessary the classification of every subpopulation before applying the model, the units of measure and the population have great variability. That is the main reason why the comparisons presented are indirect and the generalization of the data that we show must be read carefully.

## Conclusions and recommendations for future research

This scoping review highlights the principal information of different PopPK models, which showed heterogeneity in the parameters and methods of analysis and evaluation, even if these methods can be used to guide the dosing regimen in different subpopulations, it is imperative to conduct experiments with local samples to define the best fit in the different subpopulation.

## Data availability

### Underlying data

All data underlying the results are available as part of the article and no additional source data are required.

### Extended data

Zenodo: Scoping review on Population pharmacokinetics of vancomycin in non-critically ill. https://doi.org/10.5281/zenodo.14876777 ^58^

This project contains the following underlying data:

• PkPop Vanco_non critical patients - Extended data E1 PRISMA-ScR checklist.docx
• PkPop Vanco_non critical ill patients - Extended data E2 Additional results tables.pdf
• LICENSE.txt- - Supplementary data S2: Additional results tables.pdf
• LICENSE.txt

Data are available under the terms of the Creative Commons Attribution 4.0 International license (CC-BY 4.0).
