# Supplementary material for: Vancomycin Population Pharmacokinetic Models in Non- Critically Ill Adults Patients: a scoping review
Source: F1000Res. 2025 Mar 6;11:1513. Originally published 2022 Dec 13. [Version 2] doi: 10.12688/f1000research.128260.2 (PMC11928783; doi:10.12688/f1000research.128260.2)
Supplement: Peer review history

# Reviewer report: Reviewer response for version 2

**DOI:** 10.5256/f1000research.178348.r369835

**Published:** 2025-03-21

**Authors:** Venkata Kashyap Yellepeddi

**Affiliations:** 1 Spencer Fox Eccles School of Medicine, Department of Pediatrics, The University of Utah, Salt Lake City, Utah, USA

Authors have addressed all my comments satisfactorily.

Are the rationale for, and objectives of, the Systematic Review clearly stated?

Yes

Is the statistical analysis and its interpretation appropriate?

Not applicable

If this is a Living Systematic Review, is the ‘living’ method appropriate and is the search schedule clearly defined and justified? (‘Living Systematic Review’ or a variation of this term should be included in the title.)

Not applicable

Are sufficient details of the methods and analysis provided to allow replication by others?

Yes

Are the conclusions drawn adequately supported by the results presented in the review?

Yes

Reviewer Expertise:

Clinical Pharmacology, Nanotechnology. Mode-informed drug design, Population PK modeling, Physiologically based pharmacokinetic modeling.

I confirm that I have read this submission and believe that I have an appropriate level of expertise to confirm that it is of an acceptable scientific standard.

# Reviewer report: Reviewer response for version 2

**DOI:** 10.5256/f1000research.178348.r369834

**Published:** 2025-03-20

**Authors:** Manal Abouelkheir

**Affiliations:** 1 Misr International University, Cairo, Cairo Governorate, Egypt

Thank you to the authors for revising the manuscript and incorporating all the requested modifications. I have no further comments.

Are the rationale for, and objectives of, the Systematic Review clearly stated?

Yes

Is the statistical analysis and its interpretation appropriate?

Partly

If this is a Living Systematic Review, is the ‘living’ method appropriate and is the search schedule clearly defined and justified? (‘Living Systematic Review’ or a variation of this term should be included in the title.)

Not applicable

Are sufficient details of the methods and analysis provided to allow replication by others?

Partly

Are the conclusions drawn adequately supported by the results presented in the review?

Partly

Reviewer Expertise:

Antibiotics pharmacokinetics

I confirm that I have read this submission and believe that I have an appropriate level of expertise to confirm that it is of an acceptable scientific standard.

# Reviewer report: Reviewer response for version 1

**DOI:** 10.5256/f1000research.140830.r234168

**Published:** 2024-08-06

**Authors:** Manal Abouelkheir

**Affiliations:** 1 Misr International University, Cairo, Cairo Governorate, Egypt

This scoping review is comprehensive and well-structured. It provides valuable insight into PopPK models of vancomycin in non-critically ill patients. It highlights important covariates and model parameters while pointing out the variability and limitations in existing studies. It also offers practical recommendations for future research. Below are some comments to improve the quality of the manuscript.

**Abstract:**

- Please make the abstract more concise by summarizing the search strategy and inclusion criteria in fewer words in the method, focusing the results on the most critical findings, such as the main covariates and models used, and finally, highlighting the key takeaway points briefly in the conclusion.

**Methods:**

- The method has two redundant sentences: “Articles that did not meet inclusion/exclusion criteria were removed. The selected articles were reviewed by two authors (J.-D.V. and D.N.) and compared to inclusion and exclusion criteria.” Please remove one of them.
- The research question “What is the existing evidence related to PopPK models of vancomycin in non-critical hospitalized adult patients?"  actually follows the PCC format (Population, Concept, Context) which is generally more appropriate than PICO for scoping reviews. The PICO format (Population, Intervention, Comparison, Outcome) is commonly used for formulating research questions in systematic reviews and clinical research, especially for randomized controlled trials. However, for scoping reviews, which aim to map the existing literature on a particular topic or research area and identify key concepts, gaps, and evidence types, the PICO format might not always be the best fit.
- The authors mentioned that they included studies published between January 1980 and November 2021. But I noticed that some studies are missing, for example: Population Pharmacokinetic Model for Vancomycin Used in Open Heart Surgery: Model-Based Evaluation of Standard Dosing Regimens ( https://doi.org/10.1128/aac.00088-18 ) and Dose Tailoring of Vancomycin Through Population Pharmacokinetic Modeling Among Surgical Patients in Pakistan ( https://doi.org/10.3389/fphar.2021.721819). In addition, more studies are published thereafter, like: Population pharmacokinetic model of vancomycin in postoperative neurosurgical patients ( https://doi.org/10.3389/fphar.2022.1005791). I suggest to either including them in the revised version or explaining the reason why you excluded such studies.
- Remove the sentence “The results were categorized and organized into subgroups in Table 2 and Table 3.” From the method section and better refer to these tables in the results section in a more descriptive way for the content of each table.

**Results:**

- The authors mentioned that “after removing the duplicate studies, 134 articles remained for screening and 73 of these articles were selected for review. Finally, we considered 17 articles that met the inclusion/exclusion criteria”. The stepwise screening approach needs further clarification. What is the difference between 1 ^st^ and 2 ^nd^ screening steps? Could you please further clarify on which base you reduced the number of studies from 134 to 73 and then to 17??
- It is mentioned in the results section that “The study populations were categorized into four groups: (a) renal, (b) obese, (c) older, and (d) cancer patients (Figure 1)” However, Figure 1 shows the flowchart of selected studies, not the patient categorization. Please correct it.
- The results section mentions that “these results are shown in Table 2.” A suggested better description for Table 2 would be “ A summary of demographics and PopPK modeling methods for all the included studies is presented in Table 2.”
- All abbreviations in the tables should be spelled out as a footnote under the tables.
- The results section mentions that “Only 3 studies (17.3%) had external validation (Table 3).” However, information about validity is mentioned in Table 2, not 3.
- It is mentioned in the results section “Table 3 summarizes the results of the 17 studies”. Actually table 3 summarizes the models formula, and parameters not the whole results of the 17 studies. 
- “Table 3: Characteristics PK models” Please write a more descriptive title for Table 3.
- It is mentioned that “The lowest vancomycin CL value was reported in the study by Ma KF et al., where a typical value of 2.08 L/h was reported. For the patients in this study, a mean value of 2.08mL/min was reported for CLCR.” The value 2.08 couldn’t be the same for both vancomycin Cl and CLCR. I believe it is a typo for CLCR, please correct it and rephrase the sentence for better readability.
- It is mentioned that “The lowest TVV was 42 L in the subgroup of non-cancer patients in the Alqhatani study and the highest was 66.4 L in the patients with unstable and stable kidney function.7”. Reference 7 is the updated vancomycin guideline 2020, not the study that reported this value of TVV. Also, how come the TVV of 66.4 L is the same for patients with stable and unstable kidney function? Do you mean that kidney function has no effect on TVV?
- In the result section, it is mentioned that “Although most studies with two-compartment models reported parameters in the form of flow rates (CL and Q), two studies reported model parameters in the form of elimination, transfer rate constants (k _10_, k _12_, k _21_) were presented.” Could you please spell out each abbreviation when it first appears?
- In the result section, it is mentioned that “One article used furosemide and other cystatin C for glomerular filtration rate (GFR) estimation.” How furosemide was used for GFR estimation?
- “GFR was the main covariable that affected the models, and this covariate was used to explain between-individual variability in drug clearance.” What about covariates to explain variability in volume of distribution”

**Discussion:**

- It is mentioned in the discussion section that “Despite the multiple limitations of the Cockcroft Gault equation, most of the articles used it”. Could you please elaborate more on the limitations of Cockcroft Gault equation.
- “In the volume parameter, we can notice that the two volumes that are above the mean are those of the geriatric patients and the general group.” And then you referred to the Medellin Garibay article, which actually includes trauma patients who are considered a special population, not a general population.
- In the discussion sections the authors commented only on the populations who have above the mean clearance and volume of distribution. Could you please also comment on populations who have below the mean parameters.
- In the discussion section, “For the obese subpopulation, the elimination rate constant (k10=CL/V) could be affected by the patients being overweight and in the geriatric subpopulation by a change in the central compartment volume. A solution to this problem would be to increase the loading dose to achieve a faster steady state and make it easier for them to achieve the target of AUC/MIC > 400 to ensure an adequate bactericidal effect.” This paragraph is a bit confusing. You already mentioned in a previous paragraph that obese patients had higher CL and geriatric had higher V, you just repeated the same information but in another word. And does the loading dose would be helpful only with obese and geriatric patients, or with other general populations?
- The order of the paragraphs of the discussion section needs to be revised for better readability. The 3 ^rd^ and 7 ^th^ paragraphs talk about vancomycin elimination, renal patients, and equations to estimate renal function. I suggest combining them together. Again, the 8 ^th^ paragraph can be combined with the 5 ^th^ one when talking about cancer patients.

**General comments:**

  

- The whole manuscript needs proofreading with language and grammatical editing to improve readability.
- Abbreviations should be spelled out when 1 ^st^ appears.
- Please replace the term “non-critical adults” throughout the text with “non-critical ill adults.”.

Are the rationale for, and objectives of, the Systematic Review clearly stated?

Yes

Is the statistical analysis and its interpretation appropriate?

Partly

If this is a Living Systematic Review, is the ‘living’ method appropriate and is the search schedule clearly defined and justified? (‘Living Systematic Review’ or a variation of this term should be included in the title.)

Not applicable

Are sufficient details of the methods and analysis provided to allow replication by others?

Partly

Are the conclusions drawn adequately supported by the results presented in the review?

Partly

Reviewer Expertise:

Antibiotics pharmacokinetics

I confirm that I have read this submission and believe that I have an appropriate level of expertise to confirm that it is of an acceptable scientific standard, however I have significant reservations, as outlined above.

# Response

**Published:** 2025-02-16

**Authors:** Rosa Helena Bustos

**Affiliations:** Universidad de La Sabana, Colombia

Original comments of the reviewer

 Reply by the author(s)

 Changes done on page number and line number

 This scoping review is comprehensive and well-structured. It provides valuable insight into PopPK models of vancomycin in non-critically ill patients. It highlights important covariates and model parameters while pointing out the variability and limitations in existing studies. It also offers practical recommendations for future research. Below are some comments to improve the quality of the manuscript.

 We would like to thank the reviewer for the painstaking review of our document and the suggestions made. We have taken the suggestions to heart and made the appropriate corrections and revision which have thereby strengthened and enriched our manuscript

**Abstract**

 1 Please make the abstract more concise by summarizing the search strategy and inclusion criteria in fewer words in the method, focusing the results on the most critical findings, such as the main covariates and models used, and finally, highlighting the key takeaway points briefly in the conclusion.

**Answer: **The abstract has been revised and modified, with an emphasis on focusing the results on the most critical findings, such as the main covariates and models used. Key aspects of the study have been highlighted in the conclusion.

 Page 1

 Line 11-27

**Methods**

 2 The method has two redundant sentences: “Articles that did not meet inclusion/exclusion criteria were removed. The selected articles were reviewed by two authors (J.-D.V. and D.N.) and compared to inclusion and exclusion criteria.” Please remove one of them.

**Answer: **The methods section has been thoroughly revised and modified.

 Page 2

 Line 66-85

 3 The research question “What is the existing evidence related to PopPK models of vancomycin in non-critical hospitalized adult patients?"  actually follows the PCC format (Population, Concept, Context) which is generally more appropriate than PICO for scoping reviews. The PICO format (Population, Intervention, Comparison, Outcome) is commonly used for formulating research questions in systematic reviews and clinical research, especially for randomized controlled trials. However, for scoping reviews, which aim to map the existing literature on a particular topic or research area and identify key concepts, gaps, and evidence types, the PICO format might not always be the best fit.

**Answer: **The reviewer’s observation is absolutely valid. The methods section has been thoroughly revised and updated."

 Page 2

 Line 71-73

 4 The authors mentioned that they included studies published between January 1980 and November 2021. But I noticed that some studies are missing, for example: Population Pharmacokinetic Model for Vancomycin Used in Open Heart Surgery: Model-Based Evaluation of Standard Dosing Regimens (https://doi.org/10.1128/aac.00088-18 ) and Dose Tailoring of Vancomycin Through Population Pharmacokinetic Modeling Among Surgical Patients in Pakistan (https://doi.org/10.3389/fphar.2021.721819). In addition, more studies are published thereafter, like: Population pharmacokinetic model of vancomycin in postoperative neurosurgical patients ( https://doi.org/10.3389/fphar.2022.1005791). I suggest to either including them in the revised version or explaining the reason why you excluded such studies.

**Answer: **A new literature search has been ongoing as of November 2024, leading to an increase in the number of included articles from 17 in the initial review to 36 in this revised version submitted to the reviewers. The analysis of these studies has been presented in Tables 2 and 3. Additionally, the articles suggested by the reviewer have been incorporated into the updated literature search

 Page 2 Line 76-79

 Table 2, Page 6-9

 Table 3, Page 10-13

 5 Remove the sentence “The results were categorized and organized into subgroups in Table 2 and Table 3.” From the method section and better refer to these tables in the results section in a more descriptive way for the content of each table.

**Answer: **The results section has been completely revised. The findings from the newly included articles in Tables 2 and 3 have been incorporated, and their description has been presented in this section.

 Table 2, Page 6-9

 Table 3, Page 10-13

 Page 3 Line 92-96

 Page 3 Line 122-141

 Page 4 Line 142-145

**Results**

 6 The authors mentioned that “after removing the duplicate studies, 134 articles remained for screening and 73 of these articles were selected for review. Finally, we considered 17 articles that met the inclusion/exclusion criteria”. The stepwise screening approach needs further clarification. What is the difference between 1st and 2nd screening steps? Could you please further clarify on which base you reduced the number of studies from 134 to 73 and then to 17??

**Answer: **The study flowchart has been modified to include the newly selected studies. The description of identification, screening, and inclusion has been carefully reviewed.

 Figure 1 -Page 3

 7 It is mentioned in the results section that “The study populations were categorized into four groups: (a) renal, (b) obese, (c) older, and (d) cancer patients (Figure 1)” However, Figure 1 shows the flowchart of selected studies, not the patient categorization. Please correct it.

**Answer: **The flowchart has been revised and updated to include the newly identified studies from the expanded search. However, Figure 1 does not display the categorization of the groups. The description of the categorization has been included in the results section.

 Page 2

 Line 122-125

 8 The results section mentions that “these results are shown in Table 2.” A suggested better description for Table 2 would be “ A summary of demographics and PopPK modeling methods for all the included studies is presented in Table 2.”

**Answer:** The description of Table 2, as suggested by the reviewer, has been included in the text.

 Page 4 Line 144-145

 9 All abbreviations in the tables should be spelled out as a footnote under the tables.

**Answer:** The abbreviations in the table have been spelled out under the tables.

 Table 2 Page 9

 Table 3 Page 13

 10 The results section mentions that “Only 3 studies (17.3%) had external validation (Table 3).” However, information about validity is mentioned in Table 2, not 3.

**Answer:** The results section has been revised and modified according to the reviewer’s suggestion

 Page 3 Line 92-96

 Page 3 Line 122-141

 Page 4 Line 142-145

 11 It is mentioned in the results section “Table 3 summarizes the results of the 17 studies”. Actually table 3 summarizes the models formula, and parameters not the whole results of the 17 studies.

**Answer:** The number of included articles has increased from 17 in the initial review to 36 in this revised version, which has been submitted to the reviewers. All 36 studies have been included in Tables 2 and 3. *Note: The study by Alqahtani et al. (2020) has been included in two categories: General and Cancer.*

 Table 2, Page 6-9

 Table 3, Page 10-13

 12 “Table 3: Characteristics PK models” Please write a more descriptive title for Table 3.

**Answer:** The name of table 3 has been modified.

 Table 3 Page 10

 13 It is mentioned that “The lowest vancomycin CL value was reported in the study by Ma KF et al., where a typical value of 2.08 L/h was reported. For the patients in this study, a mean value of 2.08mL/min was reported for CLCR.” The value 2.08 couldn’t be the same for both vancomycin Cl and CLCR. I believe it is a typo for CLCR, please correct it and rephrase the sentence for better readability.

**Answer:** The results section has been completely modified due to the increased number of studies included in the paper. However, after reviewing the reviewer's suggestion, the value of 2.08 L/h was confirmed to correspond to the CL value, as presented in Table 3.

 Result section

 Table 3 Page 10-13

 14 It is mentioned that “The lowest TVV was 42 L in the subgroup of non-cancer patients in the Alqhatani study and the highest was 66.4 L in the patients with unstable and stable kidney function.7”. Reference 7 is the updated vancomycin guideline 2020, not the study that reported this value of TVV. Also, how come the TVV of 66.4 L is the same for patients with stable and unstable kidney function? Do you mean that kidney function has no effect on TVV?

**Answer:**  The TVV values have been reviewed. The description of the TVV values for each category has been modified according to the newly included studies.

 Page 5

 Line 202-208

 15 In the result section, it is mentioned that “Although most studies with two-compartment models reported parameters in the form of flow rates (CL and Q), two studies reported model parameters in the form of elimination, transfer rate constants (k10, k12, k21) were presented.” Could you please spell out each abbreviation when it first appears?

**Answer: **The abbreviations have been reviewed throughout the text and spelled out upon their first appearance.

 All document

 16 In the result section, it is mentioned that “One article used furosemide and other cystatin C for glomerular filtration rate (GFR) estimation.” How furosemide was used for GFR estimation?

 The paragraph has been revised and modified.

**Answer: **Furosemide was not directly used to estimate the glomerular filtration rate (GFR). Instead, it was administered as part of a furosemide stress test, which has been proposed as a functional marker of renal reserve. This test assesses renal response to a standardized dose of furosemide and has been used to predict acute kidney injury (AKI) progression in critically ill patients. However, in the context of the referenced study, cystatin C was the primary biomarker used for GFR estimation.

 Page 4

 Line 162-167

 17 “GFR was the main covariable that affected the models, and this covariate was used to explain between-individual variability in drug clearance.” What about covariates to explain variability in volume of distribution”

 The paragraph has been revised and modified.

**Answer:** In our analysis, variability in the volume of distribution (Vd) was primarily assessed in relation to physiological and clinical covariates. While GFR significantly influenced drug clearance, other factors such as body weight, age, and serum albumin levels were evaluated as potential covariates for Vd. Among these, body weight showed the strongest correlation with Vd, which is consistent with its role in determining drug distribution, particularly for hydrophilic compounds like vancomycin.

 Table 3

 Pag 10-13

**Discussion**

 18 It is mentioned in the discussion section that “Despite the multiple limitations of the Cockcroft Gault equation, most of the articles used it”. Could you please elaborate more on the limitations of Cockcroft Gault equation.

**Answer:** A paragraph in the discussion section has been added.

 The Cockcroft-Gault equation, despite its widespread use in pharmacokinetic studies and drug dosing adjustments, has several limitations. It was developed in a specific population, primarily adult males, which limits its applicability to other groups such as elderly individuals, patients with altered body composition (e.g., obesity, cachexia), or critically ill patients. Additionally, since it relies on serum creatinine levels, it is influenced by factors such as muscle mass, diet, and hydration status, potentially leading to inaccurate estimations of renal function. The equation also lacks standardization across different creatinine assay methods, and its accuracy diminishes in patients with very low or highly fluctuating glomerular filtration rates (GFR), such as those with acute kidney injury (AKI). Moreover, the use of actual body weight can introduce further errors, particularly in patients with obesity or fluid overload. Despite these limitations, Cockcroft-Gault remains widely used due to its historical application in drug dosing guidelines and its inclusion in many pharmacokinetic models.

 Page 14

 Line 271-281

 19 “In the volume parameter, we can notice that the two volumes that are above the mean are those of the geriatric patients and the general group.” And then you referred to the Medellin Garibay article, which actually includes trauma patients who are considered a special population, not a general population.

**Answer:** We acknowledge the inconsistency in our statement regarding the classification of the population in the Medellín-Garibay study. While we initially referred to it as a "general group," we recognize that this study specifically includes trauma patients, who are considered a special population due to their distinct physiological and pharmacokinetic characteristics. The paragraph has been revised and modified.

 Table 2 and Table 3

 20 In the discussion sections the authors commented only on the populations who have above the mean clearance and volume of distribution. Could you please also comment on populations who have below the mean parameters.

**Answer: **The discussion section has been revised and a new paragraph added according to the reviewer's suggestion.

 Patients with above-the-mean vancomycin clearance and volume of distribution typically exhibit pharmacokinetic profiles associated with increased drug elimination and expanded drug distribution. Several factors may contribute to these elevated parameters, including younger age, preserved or augmented renal function, higher body weight, and conditions associated with hyperdynamic circulation, such as sepsis or burns. Higher clearance rates may result in subtherapeutic vancomycin concentrations, potentially reducing efficacy and increasing the risk of treatment failure, particularly in infections caused by less susceptible pathogens. Similarly, an increased volume of distribution may lead to lower peak concentrations, which could impact the drug’s time-dependent antibacterial activity. Given these considerations, patients with above-the-mean clearance and volume of distribution may require individualized dosing strategies, such as higher initial doses, more frequent administration, or therapeutic drug monitoring to ensure optimal target attainment while minimizing the risk of underexposure.

 Page 15

 Line 303-313

 21 In the discussion section, “For the obese subpopulation, the elimination rate constant (k10=CL/V) could be affected by the patients being overweight and in the geriatric subpopulation by a change in the central compartment volume. A solution to this problem would be to increase the loading dose to achieve a faster steady state and make it easier for them to achieve the target of AUC/MIC > 400 to ensure an adequate bactericidal effect.” This paragraph is a bit confusing. You already mentioned in a previous paragraph that obese patients had higher CL and geriatric had higher V, you just repeated the same information but in another word. And does the loading dose would be helpful only with obese and geriatric patients, or with other general populations?

**Answer**: The order of the paragraphs of the discussion section needs to be revised for better readability. The 3rd and 7th paragraphs talk about vancomycin elimination, renal patients, and equations to estimate renal function. I suggest combining them together. Again, the 8th paragraph can be combined with the 5th one when talking about cancer patients.

 Due to changes in the discussion section following the inclusion of new studies, we are unable to merge these paragraphs. However, a new paragraph has been written in the discussion section specifically addressing the findings related to elimination.

 Pag 14-15

 Line 287-303

**General comments**

 21 The whole manuscript needs proofreading with language and grammatical editing to improve readability.

**Answer: **The manuscript has been fully reviewed, focusing on language and grammatical editing to enhance readability and ensure clarity, coherence, and precision in the text.

 All document

 22 Abbreviations should be spelled out when 1st appears.

**Answer:** The abbreviations have been reviewed throughout the text and spelled out upon their first appearance.

 All document

 23 Please replace the term “non-critical adults” throughout the text with “non-critical ill adults.”.

**Answer:** The entire document has been reviewed, and the term "non-critical adults" has been included

 All document

# Reviewer report: Reviewer response for version 1

**DOI:** 10.5256/f1000research.140830.r283849

**Published:** 2024-07-18

**Authors:** Venkata Kashyap Yellepeddi

**Affiliations:** 1 Spencer Fox Eccles School of Medicine, Department of Pediatrics, The University of Utah, Salt Lake City, Utah, USA

The manuscript reports the scoping review of the PoPK models of vancomycin. The review includes details about model information, covariates assessed, and PK parameters reported. Below are some of the comments that would improve the quality of this scoping review:

 1. In the introduction, paragraph 2 must be rewritten. In its current form, its incoherent and incomplete. Please add more details with citations about the importance of PoPK modeling in finding optimal drug dosing regimens for narrow therapeutic index drugs.  The sentence "Sex had no influence

 in the variability of PK parameters" is totally out of place remove it.

 2. Please add the details of Rayyan software, such as manufacturer and country of origin.

 3. Please extend the search to the year 2024 and update the current list with new articles.

Are the rationale for, and objectives of, the Systematic Review clearly stated?

Yes

Is the statistical analysis and its interpretation appropriate?

Not applicable

If this is a Living Systematic Review, is the ‘living’ method appropriate and is the search schedule clearly defined and justified? (‘Living Systematic Review’ or a variation of this term should be included in the title.)

Not applicable

Are sufficient details of the methods and analysis provided to allow replication by others?

Yes

Are the conclusions drawn adequately supported by the results presented in the review?

Yes

Reviewer Expertise:

Clinical Pharmacology, Nanotechnology. Mode-informed drug design, Population PK modeling, Physiologically based pharmacokinetic modeling.

I confirm that I have read this submission and believe that I have an appropriate level of expertise to confirm that it is of an acceptable scientific standard, however I have significant reservations, as outlined above.

# Response

**Published:** 2025-02-16

**Authors:** Rosa Helena Bustos

**Affiliations:** Universidad de La Sabana, Colombia

We would like to thank the reviewer for the painstaking review of our document and the suggestions made. We have taken the suggestions to heart and made the appropriate corrections and revision which have thereby strengthened and enriched our manuscript

**1 In the introduction, paragraph 2 must be rewritten. In its current form, its incoherent and incomplete. Please add more details with citations about the importance of PoPK modeling in finding optimal drug dosing regimens for narrow therapeutic index drugs. The sentence "Sex had no influence.**

**Answer:** The entire introduction has been revised and updated. The reviewer’s suggestions have been incorporated, with greater emphasis placed on the importance of PopPK modeling in optimizing drug dosing regimens for narrow therapeutic index drugs

 Page 1

 Line 47-53

**2 Please add the details of Rayyan software, such as manufacturer and country of origin.**

**Answer:** The details of Rayyan have been included.

 Page 2

 Line 80-83

**3 Please extend the search to the year 2024 and update the current list with new articles.**

**Answer:** A new literature search was conducted as of November 2024. The number of included articles has increased from 17 in the initial review to 36 in this revised version, which has been submitted to the reviewers. Tables 1 and 2 present the analysis of these studies.

 Page 2

 Line 76-79

 Table 2, Page 6-9

 Table 3, Page 10-13
